# KDM4B enhances immune surveillance via demethylating cGAS

**DOI:** 10.1038/s41419-025-07792-w

**Published:** 2025-07-01

**Authors:** Qiao Peng, Huimin Zhuo, Minkang Wu, Yun Hao, Yiyi Zhang, Yuying Zheng, Lei Yu, Lin Han, Hui Ren, Yingcong Wang, Zhijie Gao, Leilei Wu, Qi Lin, Chunhua Lu, Jinghua Li, Ping Wang, Lan Fang, Haihong Yu, Meiling Lu

**Affiliations:** 1 Tongji University Cancer Center, Shanghai Tenth People’s Hospital, School of Medicine, Tongji University, Shanghai, China; 2 Department of Thoracic Surgery, Tongji Hospital, Tongji Medical College, Huazhong University of Science and Technology, Wuhan, Hubei China; 3 Shanghai Tenth People’s Hospital, School of Medicine, Tongji University, Shanghai, China; 4 Department of Thoracic Surgery, Zhejiang Cancer Hospital, Hangzhou Institute of Medicine (HIM), Chinese Academy of Sciences, Hangzhou, China; 5 College of Traditional Chinese Medicine and Pharmacy, Jinggangshan University, Ji’an, China

**Keywords:** Post-translational modifications, Immunological disorders, Tumour immunology

## Abstract

Cyclic GMP–AMP synthase (cGAS) serves as a crucial sentinel in innate immunity by sensing cytosolic DNA, yet the molecular mechanisms governing its activation remain incompletely understood. Here, we identify lysine demethylase 4B (KDM4B) as the specific demethylase that erases cGAS K350 methylation, facilitating its chromatin release and subsequent activation. Genetic ablation of *Kdm4b* compromised both antiviral immunity against HSV-1 infection and antitumor responses, while also diminishing the efficacy of anti-PD-1 immunotherapy. Mechanistically, KDM4B-mediated cGAS demethylation proved crucial for its proper subcellular distribution and activation. In the context of autoimmune diseases, we found that targeting KDM4B–cGAS axis through either genetic approaches or pharmacological inhibition of KDM4B with JIB-04 effectively ameliorated disease manifestations in both *Trex1*-deficient mice and peripheral blood mononuclear cells from Aicardi-Goutieres syndrome (AGS) patients. Collectively, this study demonstrated that KDM4B functions as a specific demethylase for cGAS, controlling its chromatin dissociation and subsequent activation, thereby providing a therapeutic rationale for targeting cGAS methylation in human diseases.

## Introduction

The maintenance of organismal homeostasis relies critically on the rapid detection and elimination of potential threats. Among various danger signals, cytosolic DNA serves as a crucial indicator of pathogenic invasion or cellular dysfunction, which is primarily detected by cyclic GMP–AMP synthase (cGAS), a key sentinel of innate immunity [1]. Upon sensing double-stranded DNA (dsDNA), cGAS catalyzes the synthesis of cyclic GMP–AMP (cGAMP), which activates the stimulator of interferon genes (STING) [2], triggering a signaling cascade through TANK-binding kinase 1 (TBK1) and interferon regulatory factor 3 (IRF3) to induce type I interferon production [3, 4]. While this cGAS–STING pathway is essential for antitumor immunity and antiviral defense [5, 6], its aberrant activation by self-DNA can trigger excessive interferon production, potentially leading to autoimmune disorders such as Aicardi–Goutieres syndrome (AGS) and systemic lupus erythematosus (SLE) [7, 8], highlighting the critical importance of precise regulation of cGAS activity.

Multiple post-translational modifications (PTMs), including ubiquitination, phosphorylation, acetylation, lactylation and methylation, orchestrate the intricate control of cGAS activation and function [1, 9, 10]. Notably, protein arginine methyltransferases PRMT1 and PRMT5 methylate cGAS, significantly impacting cGAS-mediated antitumor and antiviral responses [11, 12]. The subcellular localization of cGAS adds another layer of complexity to its regulation. During homeostasis, cytoplasmic exposure of chromatin during mitosis recruits cGAS to nucleosomes, where it becomes chelated and self-inactivated to prevent inappropriate dsDNA binding [13, 14]. This nuclear sequestration is dynamically regulated. CRL5-SPSB3 ubiquitin ligase targets nuclear cGAS for degradation upon mitotic exit, and interference with SPSB3-mediated nuclear cGAS degradation enhances interferon production and antiviral immunity [15]. Furthermore, our previous work demonstrated that methylation of cGAS at K362 (human)/K350 (mouse) by SUV39H1 promotes its nuclear localization and nucleosome binding, while blocking this methylation through methionine restriction enhances cGAS activity and antitumor immunity [16]. However, the mechanism governing chromatin release and reactivation of methylated cGAS remains elusive.

KDM4B, a member of the lysine demethylase 4 (KDM4/JMJD2) family [17, 18], has emerged as a crucial regulator in various pathological conditions, including cancer, aging, and obesity [19–21]. Beyond its well-characterized role in H3K9me3 and H3K36me3 demethylation [22–24], KDM4B physically interacts with non-histone proteins, such as TRAF6, c-MYC and c-Jun to regulate their activities in tumorigenesis depending on its demethylase activity [21, 25, 26]. However, its potential role in immune surveillance remains unexplored.

In this study, we identify KDM4B as a critical regulator of cGAS activity through K350 demethylation, facilitating its release from chromatin and subsequent activation. We demonstrate that *Kdm4b* deficiency compromises both antiviral and antitumor immunity. Importantly, we show that targeting cGAS demethylation, either through K350R mutation or KDM4B inhibition with JIB-04, ameliorates autoimmune manifestations in both *Trex1*^*−/−*^ mice and AGS patient PBMCs, presenting a promising therapeutic strategy for self-DNA-induced autoimmune disorders.

## Results

### Inhibitors screening reveals KDM4B as the specific demethylase for cGAS K350 demethylation

**Fig. 1:**
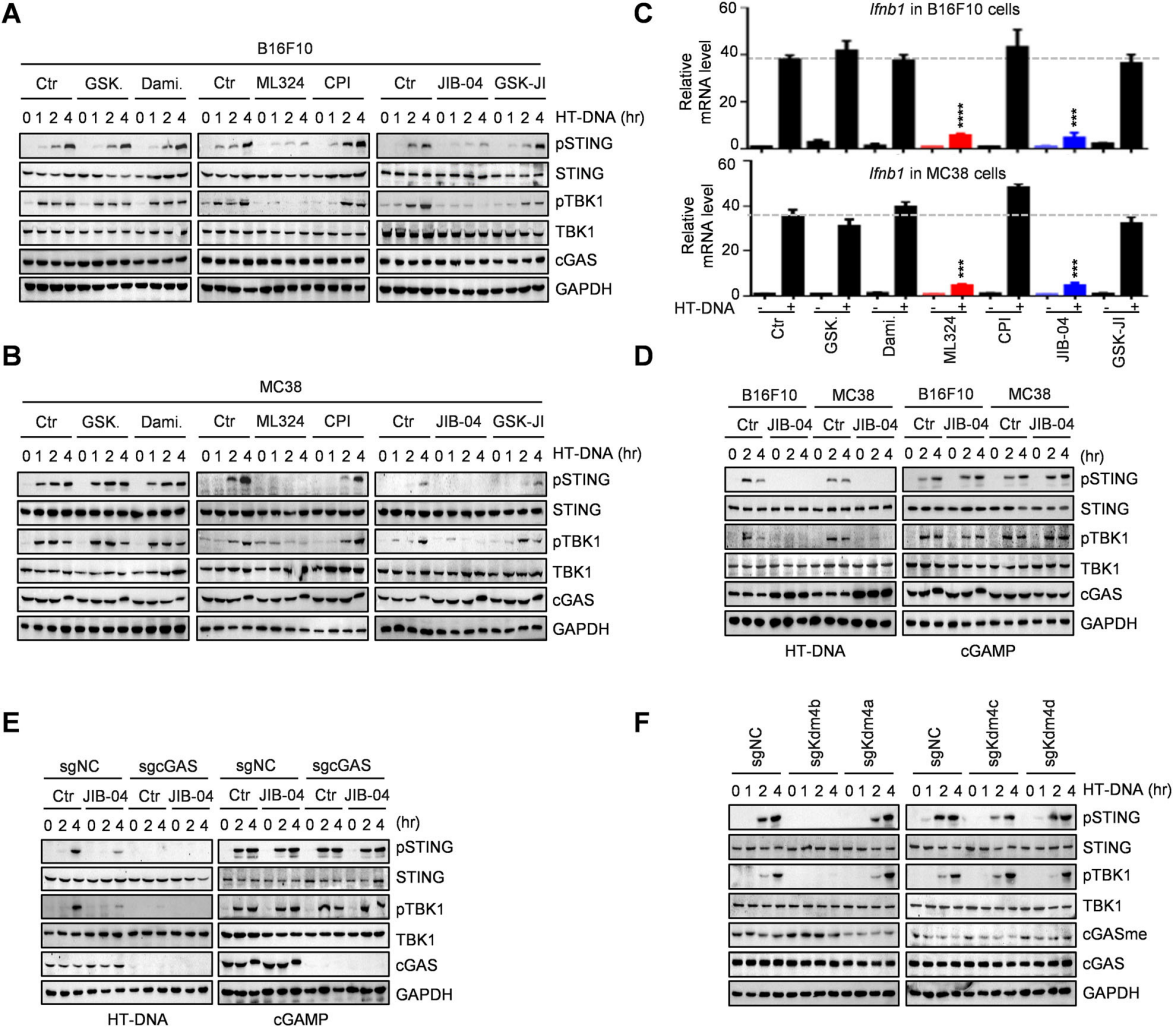
Inhibitors screening reveals KDM4B as the specific demethylase for cGAS K350 demethylation. **A–C** Effects of six demethyltransferase family inhibitors treatment on cGAS-STING activity (**A**, **B**) and *Ifnb1* expression (**C**) in B16F10 and MC38 cells. Cells were pre-treated with 1 μM GSK-LSD1 (GSK.), 2 μM Daminozide (Dami.), 50 μM ML324, 25 μM CPI-455 (CPI.), 20 μM JIB-04, and 20 μM GSK-J1 for 12 h, followed by HT-DNA stimulation. **D** Effects of JIB-04 treatment on cGAS–STING activity in B16F10 and MC38 cells with or without HT-DNA or cGAMP stimulation. Cells were treated with 20 μM JIB-04 for 12 h. **E** Effects of JIB-04 treatment on cGAS–STING activity in B16F10 *Cgas* knockout (KO) and control cells with or without HT-DNA or cGAMP stimulation. Cells were treated with 20 μM JIB-04 for 12 h. **F** cGAS–STING activity in B16F10 *Kdm4a* KO, *Kdm4b* KO, *Kdm4c* KO, *Kdm4d* KO, and control cells. Cells were treated with HT-DNA (1 μg/ml) for the indicated hours. Data are mean ± SEM for **B**, one-way ANOVA. ***P < 0.001, ****P < 0.0001.

Aberrant accumulation of self-DNA in the cytosol can lead to the production of interferons (IFN) and the onset of autoimmune and inflammatory diseases. Given that cGAS methylation plays a crucial role in its activation and function, we aimed to identify the specific demethylase that erases cGAS methylation and regulates its activity. We screened inhibitors targeting the six major lysine demethylase families (KDMs) and found that treatment with KDM4 inhibitors ML324 and JIB-04 significantly suppressed cGAS–STING activity, as evidenced by the decreased phosphorylated levels of STING (pSTING) and TBK1 (pTBK1) as well as reduced IFN production (Fig. 1A–C). Importantly, JIB-04 suppressed cGAS–STING activation induced by HT-DNA treatment but not by cGAMP (Figs. 1D and S1A, B), indicating that JIB-04 inhibits HT-DNA-induced cGAS–STING activation via regulating cGAS. Furthermore, knockout of *Cgas* abolished the effect of JIB-04 treatment (Fig. 1E). These findings suggest that the KDM4 histone demethylase subfamily is involved in cGAS demethylation.

The mouse KDM4 subfamily consists of KDM4A, KDM4B, KDM4C, and KDM4D. To determine which demethylase specifically erases cGAS K350 methylation, we generated individual knockout *Kdm4a*, *Kdm4b*, *Kdm4c*, and *Kdm4d* cell lines. Notably, knockout of *Kdm4b*, but not other KDM4 family members, significantly inactivated cGAS–STING signaling and increased cGAS methylation at the K350 site (Fig. 1F). These results identified KDM4B as the specific demethylase targeting cGAS K350 methylation.

### KDM4B activates cGAS–STING signaling through untethering cGAS from chromatin

**Fig. 2:**
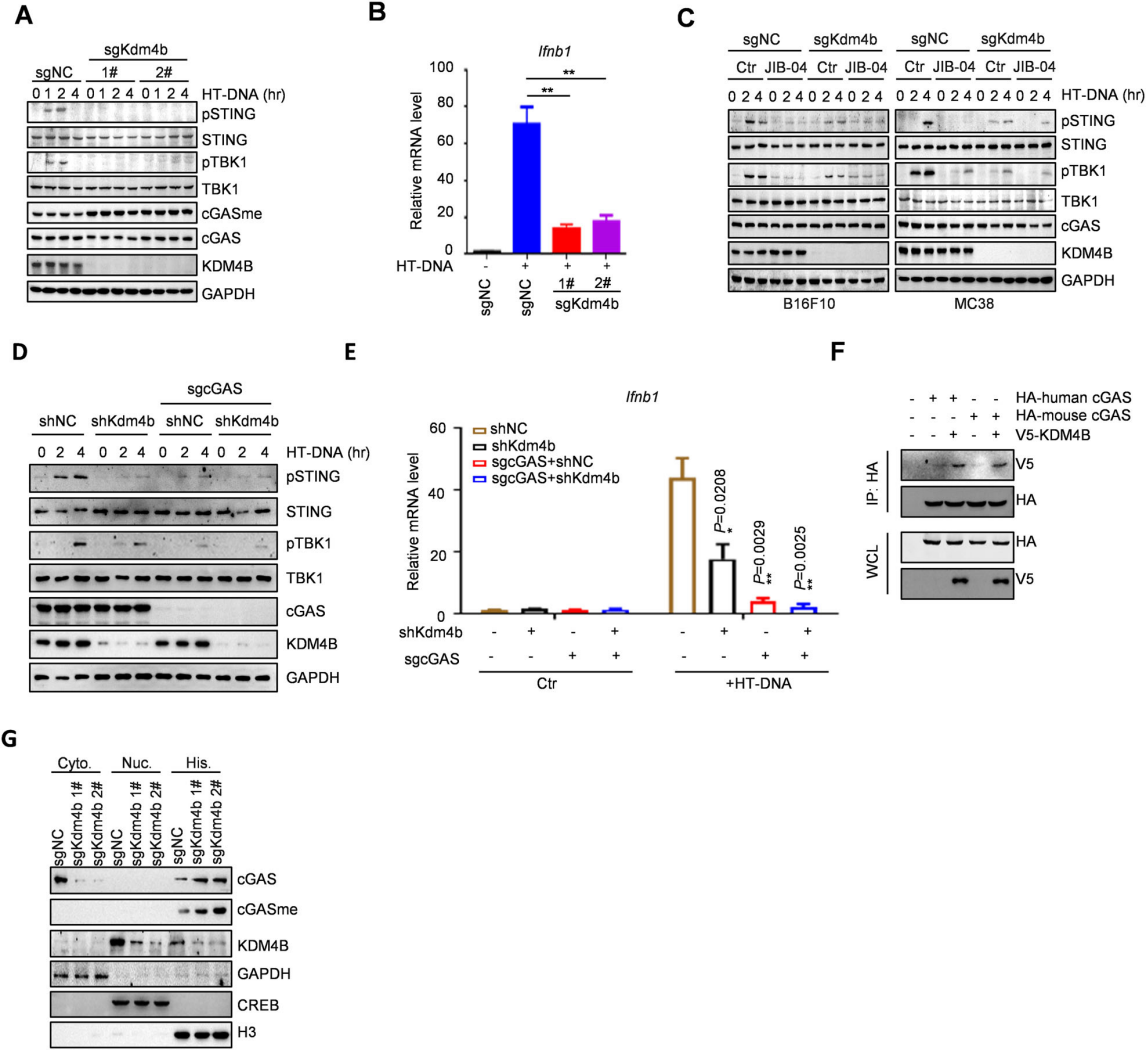
KDM4B activates cGAS–STING signaling through untethering cGAS from chromatin. **A** cGAS–STING activity in two individual B16F10 *Kdm4b* KO and control cells. Cells were treated with HT-DNA (1 μg/ml) for the indicated hours. **B** *Ifnb1* expression in *Kdm4b* KO and control B16F10 cells. *Ifnb1* expression was detected by HT-DNA (1 μg/ml) stimulation for 6 h. **C** Effects of JIB-04 treatment on cGAS–STING activity in B16F10 and MC38 *Kdm4b* KO and control cells. Cells were treated with 20 μM JIB-04 for 12 h. HT-DNA (1 μg/ml) or cGAMP (1 μg/ml) were treated for the indicated hours. **D** cGAS–STING activity in *Kdm4b* KO or/and *Cgas* knockout B16F10 cells. Cells were treated with HT-DNA (1 μg/ml) for the indicated hours. **E** *Ifnb1* expression in *Kdm4b* KO or/and *Cgas* knockout B16F10 cells. *Ifnb1* expression was detected by HT-DNA (1 μg/ml) stimulation for 6 h. **F** The co-immunoprecipitation of KDM4B and cGAS. The KDM4B with V5-tag was transfected into HEK293T cells with either human or mouse cGAS with HA-tag. Cell lysates were immunoprecipitated using HA antibody and protein A/G agarose beads, and then immunoblotted with the indicated antibodies. **G** Immunoblot of cGAS in the cytoplasmic, soluble and insoluble nuclear fractions from B16F10 *Kdm4b* KO cells. Data are mean ± SEM for **B** and **E**, one-way ANOVA. *P < 0.05, **P < 0.01.

To investigate the role of KDM4B in the cGAS–STING signaling pathway, we generated two different *Kdm4b* knockout cell lines, which showed that *Kdm4b* deficiency enhanced cGAS methylation and suppressed cGAS–STING signaling (Fig. 2A). *Kdm4b* knockout also reduced IFN production (Fig. 2B). Importantly, JIB-04 failed to further suppress cGAS–STING activity in *Kdm4b* knockout cells (Fig. 2C), suggesting that inhibition of KDM4B by JIB-04 inhibits cGAS activity through blocking its demethylation.

To detect whether KDM4B activates cGAS–STING signaling in a cGAS dependent manner, we generated a *Kdm4b* and *Cgas* double knockout cell line (Fig. S2). *Kdm4b* deficiency failed to further suppress cGAS–STING activity (Fig. 2D) and IFN production (Fig. 2E) in *Cgas* knockout cells. To further investigate the mechanism, we performed the co-immunoprecipitation assay in HEK293T cells and subcellular fractionation analysis in *Kdm4b* knockout cells, which showed that KDM4B binds to both human and mouse cGAS (Fig. 2F). Additionally, *Kdm4b* knockout promotes cGAS tethering to chromatin (Fig. 2G). Collectively, these results demonstrate that KDM4B demethylates cGAS and enhances cGAS untethering from chromatin, leading to the activation of the cGAS–STING pathway.

### KDM4B promotes anti-tumor response and immunotherapy through cGAS in vivo

**Fig. 3:**
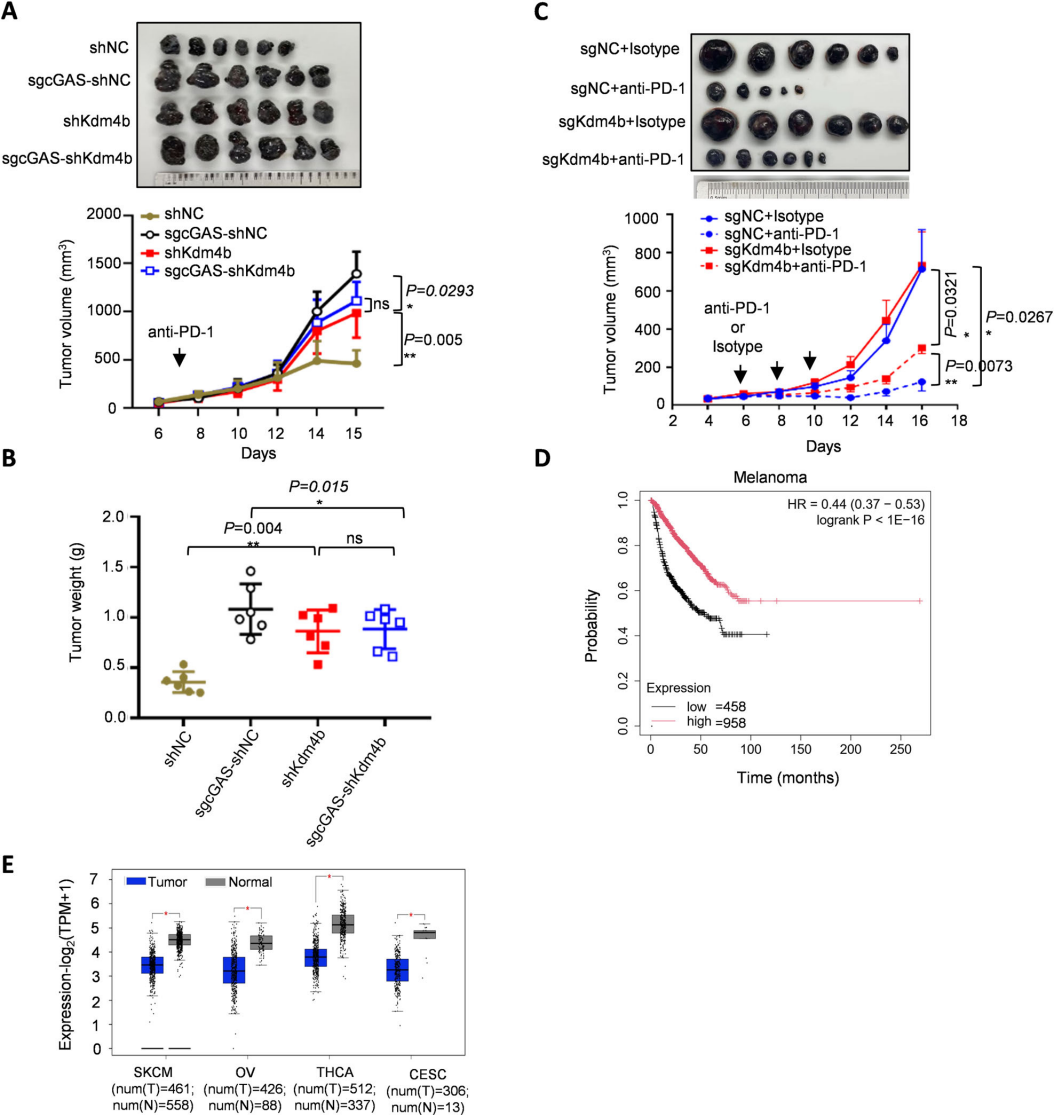
KDM4B promotes anti-tumor response and immunotherapy through cGAS in vivo*.* **A** and **B** Effects of KDM4B and cGAS double deficiency on B16F10 tumor growth (*n* = 6) analyzed by tumor picture (**A**), tumor volume (**A**), and tumor weight (**B**). **C** Effects of *Kdm4b* knockout on B16F10 tumor growth with anti-PD-1 antibody treatment (*n* = 6) displayed for tumor picture and tumor volume. **D** Kaplan–Meier analysis of overall survival in a set of 1416 melanoma samples. Statistical significance was determined by the log-rank test, *p* < 0.0001. High and low indicate the expression of KDM4B is up and down the median, respectively. **E** TCGA analysis for *Kdm4b* mRNA expression in SKCM, OV, THCA and CESE cells on the GEPIA website. Data are mean ± SEM for **A**–**C**, two-way ANOVA. *P < 0.05, **P < 0.01, ns, not significant.

Given that KDM4B serves as the demethylase of cGAS and plays a vital role in cGAS activation, we evaluated the importance of KDM4B in immune surveillance. We explored the function of the KDM4B–cGAS axis in antitumor immunity. Notably, *Kdm4b* knockout accelerated tumor progression compared to the control group, whereas *Kdm4b* deficiency failed to further increase the effect of *Cgas* knockout on tumor growth (Fig. 3A and B), suggesting that KDM4B regulates tumor progression in a cGAS-dependent manner. Since cGAS is essential for the antitumor effect of immune checkpoint blockade, we investigated whether KDM4B affects immunotherapy response. Our results showed that sgNC tumors were significantly sensitive to anti-PD-1 immunotherapy, whereas *Kdm4b* deficiency partially attenuated the effect of anti-PD-1 treatment (Fig. 3C), indicating that KDM4B might regulates immune checkpoint blockade via cGAS activation. Moreover, by analyzing data from TCGA, Kdm4b expression is significantly negative correlated with the majority of immune cell infiltration (Fig. S3A) and positive correlated with cancer prognosis (Fig. 3D) in melanoma. Furthermore, TCGA analysis also revealed that mRNA levels of KDM4B were significantly lower in various tumors compared to paired normal tissues, including skin cutaneous melanoma (SKCM), ovarian serous cystadenocarcinoma (OV), thyroid carcinoma (THCA), and cervical squamous cell carcinoma (CESC) (Fig. 3E), whereas slightly downregulated in the colorectal cancer (COAD) (Fig. S3B), suggesting that downregulated KDM4B results in cGAS inactivation and immune escape during tumor progression. Collectively, these findings demonstrate that KDM4B facilitates anti-tumor immunity and enhances the efficacy of immunotherapy by demethylating and activating cGAS.

### *Kdm4b* knockout decreases cGAS-mediated anti-virus in vivo

**Fig. 4:**
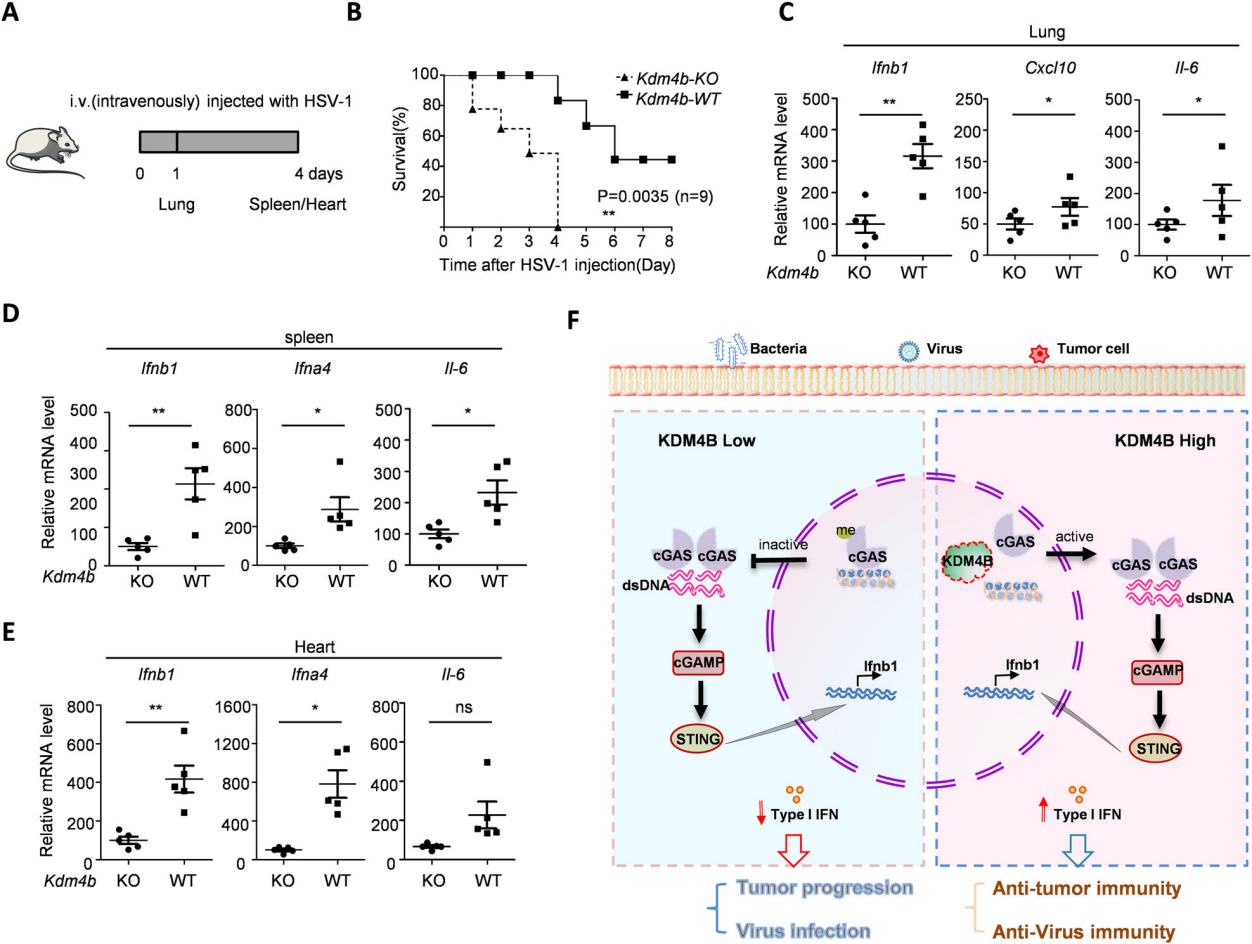
*Kdm4b* knockout decreases cGAS-mediated anti-virus in vivo*.* **A** Timeline for HSV-1 treatment in the indicated mice. **B** Kaplan–Meier (KM) analyses of the survival in the *Kdm4b* KO and WT mice treated with HSV. Mice were infected with 1 × 10^6^ pfu HSV for 4 days. The survivals were monitored for 8 days. *P* values were obtained from the log-rank test. *n* = 6 mice. **C** Type I IFN expression including *lfnb1*, *Cxcl10*, and *Il6* were detected in the lung from the *Kdm4b* KO and WT mice treated with HSV for 1 day. **D** and **E** Type I IFN expression including *lfnb1*, *lfna4* and *Il6* were detected in the spleen (**D**) and heart (**E**) from the *Kdm4b* KO and WT mice treated with HSV for 4 days. **F** Proposed working model of KDM4B on cGAS–STING signaling and related diseases. Data are mean ± SEM for **C**–**E**, unpaired *t* test. *P < 0.05, **P < 0.01, ns, not significant.

Timely defense against viral infections is a crucial aspect of immune surveillance. To evaluate the role of KDM4B in cGAS-mediated anti-viral response, we generated *Kdm4b* knockout mice and infected both *Kdm4b* wild type (WT) and knockout (KO) mice with HSV-1 (Fig. 4A). Survival analysis showed that *Kdm4b* knockout significantly decreased survival rates compared to WT mice upon intraperitoneal (IP) injection of HSV-1 (Fig. 4B). Furthermore, *Kdm4b* deficiency suppressed HSV-stimulated IFN expression in various visceral organs, including the lung (Fig. 4C), spleen (Fig. 4D), and heart (Fig. 4E), demonstrating that KDM4B promotes anti-viral immunity via regulating cGAS activity.

Our study demonstrates that KDM4B functions as the demethylase of cGAS K350 methylation. Under physiological conditions, part of cGAS is methylated and sequestered in the chromatin, maintaining it in an inactive state. Under certain conditions, such as infection with nucleic acid from virus or dead tumor cells, KDM4B demethylates cGAS, untethering it from chromatin and allowing its translocation into the cytoplasm where it becomes activated. This activation leads to anti-viral responses and anti-tumor immunity. Conversely, *Kdm4b* knockout prevents cGAS demethylation in the nucleus, promotes cGAS tethering to the chromatin, and inhibits cGAS activation, thus impeding immune surveillance (Fig. 4F).

### KDM4B inhibition blocks the hyperactivation of cGAS in autoimmune disease

**Fig. 5:**
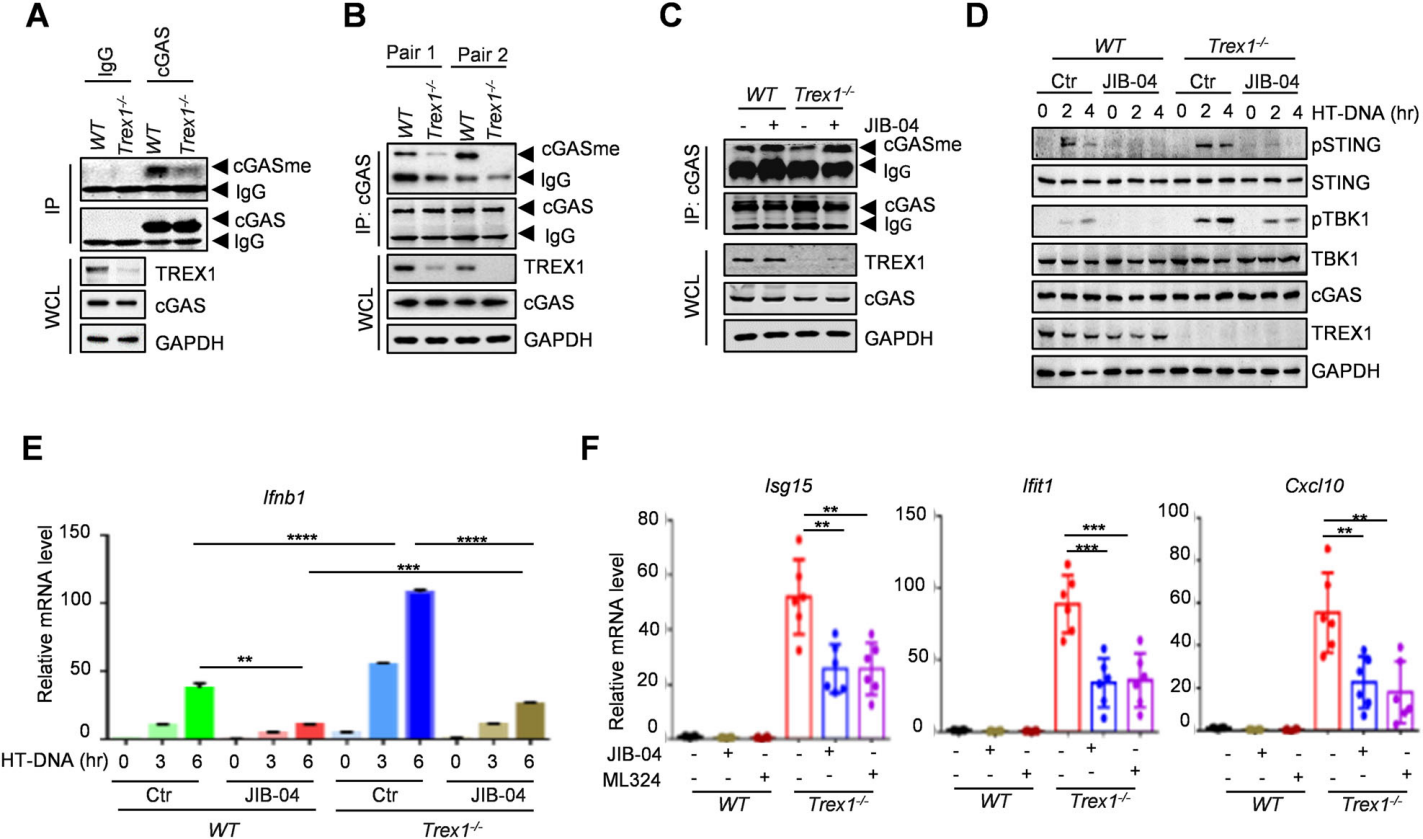
KDM4B inhibition blocks the hyperactivation of cGAS in autoimmune disease. **A** Immunoblot of endogenous methylated cGAS in bone marrow cells from *Trex1*^*−/−*^ and wild-type (WT) mice. **B** Immunoblot of endogenous methylated cGAS in spleen cells from *Trex1*^*−/−*^ and wild-type (WT) mice. **C**–**E** Effects of 20 μM JIB-04 treatment on cGAS methylation (**C**), cGAS-STING activity (**D**), and *Ifnb1* expression (**E**) in bone marrow cells from *Trex1*^*−/−*^ and wild-type (WT) mice. **F** ISGs expression in bone marrow cells from *Trex1*^*−/−*^ and wild-type (WT) mice treated with 20 μM JIB-04 or 20 μM ML324 for 2 days. Data are mean ± SEM for **E** and **F**, one-way ANOVA. **P < 0.01, ***P < 0.001, ****P < 0.0001.

Uncontrolled activation of cGAS is one of the major causes of autoimmune diseases, manifesting as an overreaction of immune surveillance. We explored whether targeting cGAS methylation could be a viable strategy for treating self-DNA-induced autoimmune diseases. TREX1, a cytoplasmic deoxyribonuclease, is the major 3’–5’ exonuclease in mammalian cells that degrades cytoplasmic dsDNA and prevents cGAMP synthesis by cGAS. Mutations in TREX1 have been identified in patients with Aicardi-Goutières syndrome (AGS) [27, 28], where accumulated cytoplasmic self-DNAs chronically activate cGAS-mediated type I IFN production, driving systemic inflammation and autoimmune disorders. Notably, we found that cGAS methylation was significantly decreased in bone marrow (BMDM) and spleen cells isolated from *Trex1*^*−/−*^ mice compared with wild-type mice (Fig. Fig 5), implying that cGAS/STING signaling was high activated in *Trex1*^*−/−*^ mice. To determine whether blocking demethylation of cGAS suppresses self-DNA-induced autoimmunity, BMDM cells isolated from both wild-type and *Trex1*^*−/−*^ mice were treated with the KDM4B inhibitor JIB-04. JIB-04 treatment increased endogenous cGAS methylation, suppressed cGAS–STING activity, and reduced IFN production in *Trex1*^*−/−*^ BMDM cells (Fig. 5C–E). Furthermore, treatment with KDM4B inhibitors JIB-04 or ML324 inhibited the expression of ISGs in *Trex1*^*−/−*^ BMDM cells (Fig. 5F). Conversely, methionine starvation reduced cGAS methylation, and strongly promoted cGAS–STING activity and IFN production in bone marrow cells from Trex1^−/−^ mice (Fig. S4A–C). The expression of ISGs in *Trex1*^*−/−*^ BMDM cells was also strongly increased by methionine starvation and chaetocin treatment (Fig. S4D). These data suggest that inhibition of KDM4B promotes cGAS methylation and subsequent inactivation in *Trex1*^*−/−*^ mice, which could potentially block the overactivation of cGAS–STING signaling and benefit autoimmune disease management.

### K350 is sufficient for cGAS activity in autoimmune disease

**Fig. 6:**
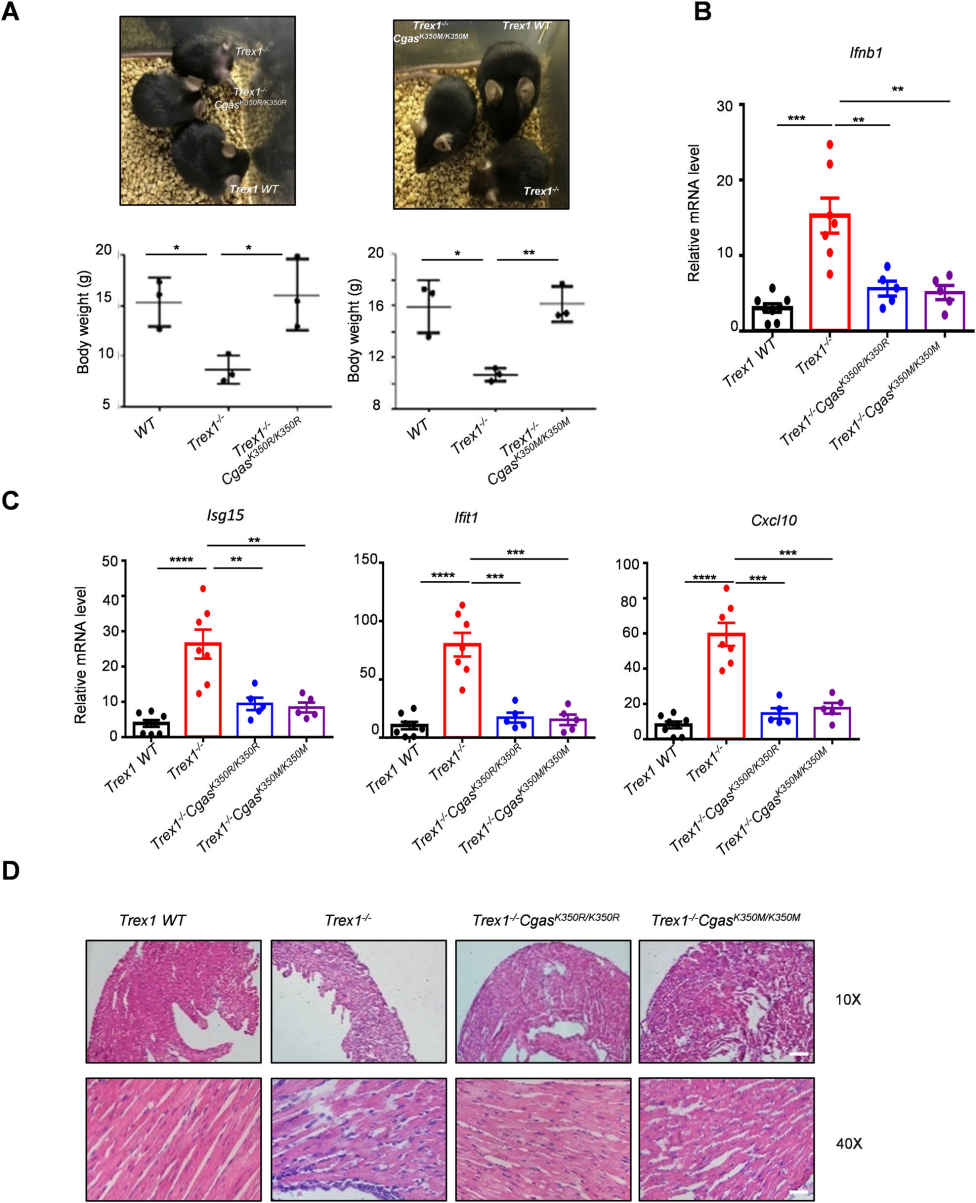
K350 is sufficient for cGAS activity in autoimmune disease. **A** Picture and body weight of wild-type (WT), *Trex1*^*−/−*^, and *Trex1*^*−/−*^*Cgas*^*K350R/K350R*^ littermate mice (left), and wild-type (*WT*), *Trex1*^*−/−*^, and *Trex1*^*−/−*^*Cgas*^*K350M/K350M*^ littermate mice (right) (*n* = 3). **B**, **C** *Ifnb1* (**B**) and ISGs (**C**) expression in hearts of mice of the indicated genotype. **D** Paraffin-embedded heart tissue sections from mice of the indicated genotype. The top panels are at ×10 and the bottom panels are at ×40 magnification. Data are mean ± SEM for A, unpaired *t* test. Data are mean ± SEM for **B** and **C**, one-way ANOVA. *P < 0.05, **P < 0.01, ***P < 0.001, ****P < 0.0001.

Since KDM4B specifically demethylates cGAS at K350, and *Kdm4b* knockout inactivates cGAS in autoimmune disease, we investigated the significance of cGAS K350 methylation in self-DNA-induced autoimmunity in vivo. We bred *Trex1*-deficient mice with *Cgas* K350R or K350M mice, as *Trex1-*deficient mice exhibit a self-DNA-induced autoinflammatory phenotype [28, 29]. Remarkably, *Cgas* K350R or K350M mutations prevented disease progression in *Trex1*-deficient mice, as evidenced by complete rescue from mortality and restoration of normal skin color and body weight (Fig. 6A). *Trex1*-deficient mice typically died of circulatory failure caused by severe inflammatory myocarditis [30]. Hearts from *Trex1*-deficient mice exhibited profound inflammation with high IFN and ISGs production, thinning of the ventricular wall, mononuclear cell infiltrates, and disruption of normal heart muscle morphology (Fig. 6B–D). Notably, all these pathological features were absent in *Trex1*^*−/−*^*Cgas*^*K350R/K350R*^ and *Trex1*^*−/−*^*Cgas*^*K350M/K350M*^ littermates (Fig. 6B–D). These data demonstrate that cGAS K350 methylation is a potential therapeutic target for addressing self-DNA-induced autoimmunity.

### Targeting KDM4B-mediated cGAS K350 demethylation contributes to autoimmune disease treatment

**Fig. 7:**
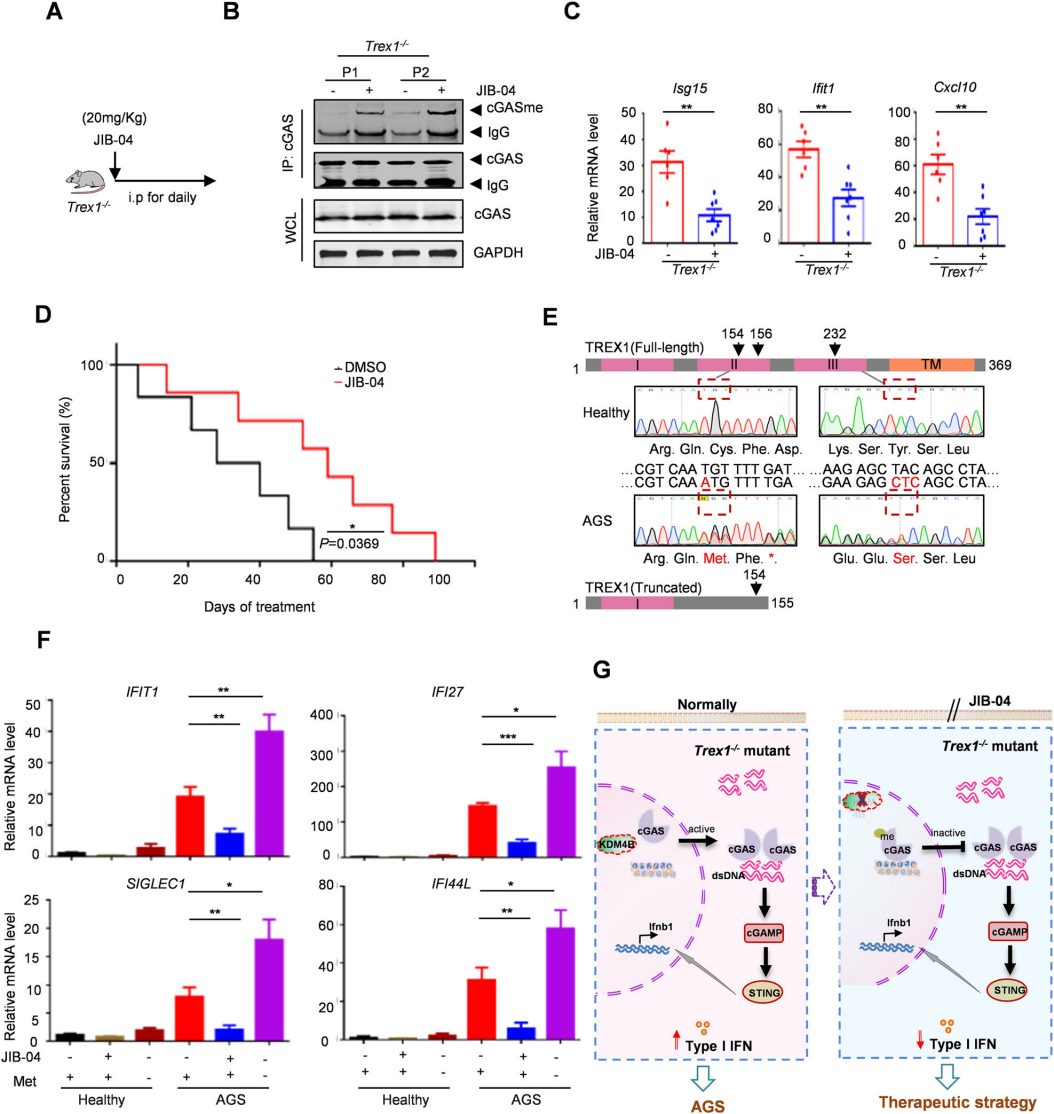
Targeting KDM4B-mediated cGAS K350 demethylation contributes to autoimmune disease treatment. **A** Experimental design in *Trex1*^*−/−*^ mice. 3 weeks old *Trex1*^*−/−*^ mice were given daily treatment of JIB-04 (20 mg/kg) or DMSO. **B**, **C** Immunoblot of endogenous methylated cGAS in spleen cells (**B**) and ISGs expression in the heart (**C**) of *Trex1*^*−/−*^ mice treated with JIB-04 or DMSO for 8 days (*n* = 7). **D** Overall survival of *Trex1*^*−/−*^ mice treated with JIB-04 or DMSO for over 3 months (*n* = 7). *P* values were obtained from the log-rank test. **E** Schematic diagram and DNA sequencing showing frameshift mutations on *TREX1* in the AGS patient in comparison with healthy person. **F** ISGs expression in PBMCs treated with DMSO, 20 μM JIB-04, or methionine starvation for 12 h. PBMCs were from the AGS patient and healthy person. **G** The working model of KDM4B on autoimmune diseases. Data are mean ± SEM for **C** and **F**, one-way ANOVA. *P < 0.05, **P < 0.01, ***P < 0.001.

To evaluate the potential therapeutic effect of KDM4B inhibitor JIB-04 in alleviating the disease phenotype, Trex1^−/−^ mice were administered JIB-04 via daily intraperitoneal injections as shown in Fig. 7A. JIB-04 treatment increased cGAS methylation in spleen cells and reduced ISGs expression in hearts of *Trex1*^*−/−*^ mice (Fig. 7B, C). Importantly, the survival time of *Trex1*^*−/−*^ mice was significantly extended by JIB-04 treatment (Fig. 7D). Thus, these data suggest that blockade of cGAS demethylation exerts an inhibitory effect on self-DNA-induced autoimmune responses in *Trex1*^*−/−*^ mice.

To examine whether methylation-mediated cGAS autoinhibition presents a therapeutic strategy for AGS, we isolated PBMCs from an AGS patient with frameshift mutations c.459dupA and c.695delA on *TREX1*, resulting in heterozygous Cys154Met and Tyr232Ser mutations, leading to highly elevated lSGs expression (Fig. 7E, F). Compared to PBMCs from healthy individuals, ISGs levels were significantly increased in the AGS patient’s PBMCs, suggesting cGAS activation by accumulated self-DNA. Notably, JIB-04 treatment markedly reduced ISGs expression in AGS patient’s PBMCs, while methionine starvation treatment promoted the levels of ISGs (Fig. 7F).

Our data demonstrate that double-stranded DNA accumulates in the cytosol in autoimmune disease, leading to cGAS overactivation and elevated IFN production. Upon *Kdm4b* knockout or inhibition with JIB-04, methylation of cGAS increases, resulting in cGAS nuclear sequestration and inactivation. This approach provides a promising therapeutic strategy for treatment of self-DNA-induced autoimmune diseases (Fig. 7G).

## Discussion

**Fig. 8:**
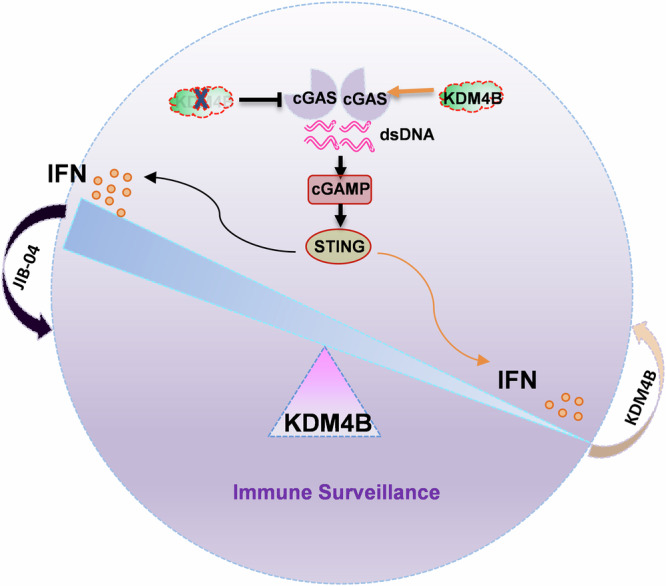
Working model depicting how KDM4B governs immune surveillance via balancing IFN production through dynamic activation of cGAS–STING signaling, and providing an effective strategy for cancer, infection and autoimmune diseases treatment.

cGAS, as the primary cytosolic DNA sensor, plays a crucial role in immune surveillance by binding to dsDNA and activating innate immune IFN responses [31, 32]. Our study reveals a key epigenetic mechanism regulating cGAS activity, demonstrating that the methylation status at K350 serves as a molecular switch controlling cGAS activation (Fig. 8). Our data show that following viral and vitro DNA infection, KDM4B demethylates cGAS at the K350 site, which is methylated by SUV39H1, thereby releasing cGAS from chromatin. This dynamic methylation regulatory mechanism not only precisely controls interferon production levels but also provides a molecular basis for rapid response to viral infection and DNA damage. Given that cGAS hyperactivation can trigger autoimmune diseases such as AGS, we found that KDM4B deficiency or its inhibitor JIB-04 significantly suppresses cGAS activation and IFN expression, providing a novel therapeutic target and strategy for autoimmune diseases.

Our findings both corroborate and extend current understanding of cGAS regulatory mechanisms. Although cGAS is typically activated by cytosolic dsDNA, it can also enter the nucleus during mitosis, where it remains inactive through chromosome binding and is subsequently degraded by the CRL5-SPSB3 system post-mitosis [15]. This dynamic nuclear-cytoplasmic distribution is essential for maintaining normal immune surveillance. Furthermore, CDK1 phosphorylates cGAS and inhibits its cGAMP synthesis during mitotic entry, while protein phosphatase 1 (PP1) dephosphorylates cGAS during mitotic exit, enabling its DNA sensing capability. This ensures appropriate host defense against cytosolic DNA during interphase while maintaining inertness to self-DNA during mitosis [33]. Our study is the first to reveal the crucial role of epigenetic modifications in this process, complementing existing phosphorylation-based regulatory mechanisms. Previous research has shown that chromatin-bound cGAS dissociates and accumulates in the nuclear soluble fraction during DNA virus infection [34], but the regulatory mechanism remained unclear. Our study reveals the molecular mechanism by which KDM4B mediates cGAS dissociation from chromatin through specific demethylation, providing a mechanistic explanation for cGAS dynamics during HSV-1 infection. Additionally, the DNA double-strand break sensor MRE11 promotes cGAS displacement from chromatin, thereby activating cGAS in response to oncogenic stress, cytosolic dsDNA, and ionizing radiation [35]. Our research suggests that KDM4B may be a key molecule linking DNA damage response and innate immune activation, offering new insights into DNA damage-induced immune responses.

More importantly, the KDM4B–cGAS regulatory axis revealed in this study has significant clinical translation value in tumor immunity and autoimmune diseases. As a key component of innate immunity, cGAS detects aberrant dsDNA to activate IFN production, which in turn promotes the maturation and mobilization of immune cells such as macrophages and natural killer (NK) cells, leading to adaptive immune responses [36, 37]. In tumor immunity, our previous work demonstrated that the SUV39H1–UHRF1 complex promotes cGAS chromatin association and suppresses its activity through methylation [16]. Moreover, a high level of methylated cGAS was associated with tumor poor outcomes, and K350R or K350M cGAS, in *Cgas* knockout tumors, cannot rescue tumor growth and immune infiltration with radiotherapy, indicating the pivotal role of K350 methylation on cGAS activity and function. The present study further identifies KDM4B as a crucial demethylase that enhances cGAS activity by removing its methylation modification, thereby promoting anti-tumor immune responses. Notably, TCGA data suggests that KDM4B is downregulated in multiple human cancers, negative correlated with the majority of immune cell infiltration but positive correlated with cancer prognosis in melanoma, indicating that tumors may evade immune surveillance by suppressing the KDM4B–cGAS signaling pathway. This finding not only reveals a novel mechanism of tumor immune evasion but also provides a theoretical foundation for developing new cancer immunotherapy strategies. However, as indicated in Fig. 3C, D, *Kdm4b* knockout tumors were only partially resistant to anti-PD-1 immunotherapy. Because of other substrates, including H3K9 and H3K36, have been identified as KDM4B substrates. Furthermore, KDM4B also involves in the P53-signaling pathway, thereby influencing the progression of melanoma [38]. Currently we cannot exclude the other possibility that KDM4B regulates anti-tumor immunity through other mechanisms.

Regarding autoimmune diseases, our findings have direct clinical application prospects. Under normal physiological conditions, cellular DNases like TREX1 prevent abnormal cGAS activation by degrading cytosolic dsDNA. However, in autoimmune diseases such as AGS and SLE, TREX1 dysfunction leads to cytosolic DNA accumulation, resulting in persistent activation of the cGAS–STING pathway and excessive inflammatory responses in multi-organ [27, 39]. Hou et al. found that the autophagy receptor CCDC50 plays a crucial role in fine-tuning cGAS–STING signaling, with CCDC50 expression levels negatively correlating with IFN pathway activation and disease severity in SLE patients [40]. While previous studies showed that aspirin could alleviate autoimmune symptoms by inhibiting cGAS [9, 41], its specificity is limited. Excitingly, we found that the specific KDM4B inhibitor JIB-04 can suppress cGAS activity by maintaining its methylation status, significantly improving autoimmune pathological features in both Trex1^−/−^ mouse models and PBMCs from AGS patient. However, only one clinical AGS patient was used. To obtain more robust conclusions, future multi-center or large-scale clinical validation is required. Notably, our previous research showed that methionine restriction promotes cGAS dissociation from chromatin by blocking its methylation [16]. In the current study, we further elucidated the molecular mechanism by which KDM4B regulates cGAS activity through demethylation. Interestingly, unlike methionine restriction, KDM4B inhibition significantly reduces ISGs expression levels. This difference suggests that targeting KDM4B’s epigenetic regulation offers better specificity and therapeutic efficacy compared to methionine restriction, which broadly affects methylation metabolism, indicating KDM4B as an ideal therapeutic target for autoimmune diseases.

In conclusion, by revealing the critical regulatory mechanism of KDM4B-mediated cGAS demethylation, our study not only deepens our understanding of innate immune regulation but also provides new therapeutic strategies for various diseases. This discovery bridges epigenetic regulation with innate immune responses, pioneering a new direction in immunotherapy. Subsequent studies should further explore the role of the KDM4B–cGAS axis in other diseases and develop more specific therapeutic strategies.

## Experimental section

### Mice

Six- to eight-week-old male wild-type C57BL/6 mice were obtained from Shanghai SLAC Laboratory (China). *Trex1*^*−/−*^ C57BL/6 mice were obtained from Bo Zhong lab (Wuhan University, China). *Cgas K350R* and *K350M* knockin C57BL/6 mice and *Kdm4b* knockout mice were obtained from the Shanghai Model Organisms Center (China). *Trex1*^*−/−*^ mice were bred with *Cgas K350R* and *K350M* mice to generate *Trex1*^*−/−*^*Cgas*^*K350R/K350R*^ and *Trex1*^*−/−*^*Cgas*^*K350M/K350M*^ mice. All *Trex1*^*−/−*^, *Trex1*^*−/−*^*Cgas*^*K350R/K350R*^, and *Trex1*^*−/−*^*Cgas*^*K350M/K350M*^ mice were used at the age of 3 weeks unless specified in the text. All mice procedures were conducted under the guidelines and the institutional animal care protocol approved by the Institutional Animal Care and Use Committee at Shanghai Tenth People’s Hospital of Tongji University. In none of the experiments did tumor size surpass 2 cm in any dimension.

### Human specimens

This study was approved by the Institutional Review Board at Shanghai Tenth People’s Hospital of Tongji University. Informed consent was obtained from all patients. Plasma, and peripheral blood mononuclear cells (PBMCs) were isolated from healthy donors and AGS patients.

### Cell culture and treatment

HEK293T cells, B16F10, and MC38 were obtained from the ATCC. The cells were cultured at 37 °C in DMEM (GIBCO) containing 10% FBS (GIBCO) and 50 μg/ml penicillin/streptomycin (Invitrogen) in the incubator with 5% CO_2_. The cell lines were characterized and verified free for mycoplasma contamination. All the KDM4 subfamily inhibitors, including JIB-04 (20 μM), ML324 (50 μM), CPI-455 HCl (25 μM), GSK-LSD1 (1 μM), GSK J1 (20 μM), and Daminozide (2 μM) were added into cells for 12 h, followed by HT-DNA (1 μg/ml) stimulation for the indicated hours. The key reagents and resources used in this study were listed in Table 1.

**Table 1:** Key reagents and resources used in this study.

Reagent or resource	Source	Identifier
Anti-TREX1	Santa Cruz	Cat# sc-271870
Anti-JMJD2B (D7E6)	Cell Signaling Technology	Cat# 8639
Anti-phospho-TBK1 (Ser172) (D52C2) XP Rabbit mAb	Cell Signaling Technology	Cat# 5483
Anti-phospho-STING (Ser365) (D8F4W) Rabbit mAb	Cell Signaling Technology	Cat# 72971
Anti-STING (D2P2F) Rabbit mAb	Cell Signaling Technology	Cat# 13647
Anti-TBK1 (D1B4) Rabbit mAb	Cell Signaling Technology	Cat# 3504
Anti-GAPDH (mouse)	Proteintech	Cat# 60004-1
Anti-mouse cGAS (D3O8O)	Cell Signaling Technology	Cat# 31659
Anti-K350me Rabbit antibody	This paper	N/A
Anti-Histone H3	Cell signaling technology	Cat# 4499
Anti-CREB	Cell signaling technology	Cat# 9197
InVivoPlus anti-mouse PD-1 (CD279)	Bio X Cell	Cat# BE0146
InVivoPlus rat IgG2a isotype	Bio X Cell	Cat# BE0089
*KDM4 subfamily inhibitors*		
JIB-04	Selleck	Cat#S7281
ML324	Selleck	Cat#S7296
CPI-455 HCl	Selleck	Cat#S8287
Daminozide	Selleck	Cat#S4800
GSK-LSD1	ApexBio	Cat#1401966-69-5
GSK J1	ApexBio	Cat#1373422-53-7
*Chemicals, kit, medium*		
2’3’-cGAMP	InvivoGen	tlrl-nacga23
HT-DNA	This paper	N/A
Chaetocin	Selleck	Cat#S8068
polybrene	Sigma	Cat#107689
puromycin	Yeasen Biotech	Cat#60210ES60
HISTOPAQUE-1077	Sigma-Aldrich	Cat#10771
Subcellular Protein Fractionation kit	Thermo Fisher Scientific	Cat#78840
Amino acids l-methionine	Sangon Biotech	Cat# 63-68-3
Methionine-depleted DMEM medium	Shanghai Duoning Biotechnology	Cat# B001-003

### Generation of knockout cells

CRISPR knockout cell lines were performed using 2 distinct sgRNA via lentiviral delivery of guide RNA into cells stably expressing Cas9 (in B16F10, MC38). Short hairpin RNA was conducted into pLKO.1-shRNA vector and delivered into cells with lentivirus. Cells in 12-well plates were infected with virus in 8 μg/ml polybrene for 2 days, then treated with puromycin at 2 mg/ml for 2–4 days, and validated for knockout efficiency by immunoblot analysis. All sgRNA oligos and shRNA for generating knockout cell lines are shown in Table 2.

**Table 2:** CRISPR-Cas9 sgRNA and shRNA.

Primer for specific gene	sgRNA oligos	Primer sequence
mouse cGAS	sgRNA1	TCTCGTACCCAAGAATGCAA
mouse Kdm4b	sgRNA1	AAGCCCGCATGGTAACCATA
	sgRNA2	CTGGATCGACTATGGCAAAG
	sgRNA3	GAATGCGGGACCATCATTGA
	sgRNA4	TGTGGAAGACCACGTTTGCC
mouse Kdm4a	sgRNA1	AGCGGGATCACCATTGAGG
	sgRNA2	CAGCTGACCCCCGAGGAGG
	sgRNA3	TGTCTGGAAATACCCCAGG
mouse Kdm4c	sgRNA1	AAGCTGGGCCCTCCTGCGG
	sgRNA2	GTCTCTGCAATTTGAGGGG
	sgRNA3	AGACAGAATACCTTTACAG
mouse Kdm4d	sgRNA1	TTTCCCTATGGCTACCACG
	sgRNA2	ATTCAAGACCTATTGGAAC
	sgRNA3	AATGTGGCATAGTGATTGA
mouse shKdm4b	shRNA1	GCGCAGACAAATTAGTGTGAA
	shRNA2	GCAGACATTCTACGAGGTCAA

### Isolation of primary cells

Mouse primary BMDMs were isolated as described in a previous protocol [16]. Briefly, mice were killed by halothane inhalation, and both femurs were dissected free of adherent tissue. The marrow cells were eluted by irrigation of the inner bone with PBS. Cells were washed by centrifugation and cultured with BMDM media, which contained 10% FBS, 1× penicillin/streptomycin, and 25 ng/ml MCSF-enriched media. BMDMs were cultured for at least 7 days, with media changes every 3–4 days. For measurement of signal pathway and cytokine production in BMDMs, macrophages (1 × 10^6^ cells/well) were cultured in a 12-well plate and stimulated as indicated. For measurement of cGAS methylation in BMDMs, macrophages were cultured in 10 cm dish plate and treated as indicated.

Human primary PBMCs were isolated with HISTOPAQUE-1077 according to the manufacturer’s protocols. Cells were maintained in RPMI-1640 medium supplemented with 10% FBS and 1× penicillin/streptomycin for 2 days. For measurement of cytokine production in PBMCs, cells were cultured in a 12- well plate and treated as indicated.

### Immunoprecipitation and western blot

Cells were harvested and lysed in 50 mM Tris–HCl (pH 7.9), 300 mM NaCl, 1% Triton X-100, supplemented with protease inhibitor and phosphatase inhibitor cocktails. Protein concentrations of cell supernatants were determined using the BCA protein assay kit. For the immunoprecipitation assay, 5% whole-cell lysate was loaded as an input sample. 95% cell lysate was incubated with protein A/G agarose beads together with specific antibodies at 4 °C overnight. All samples were followed by immunoblot with indicated antibodies. Briefly, Isopyknic cell lysates were mixed with 2× SDS loading buffer and boiled for 15 min. Proteins were analyzed by 10% SDS–PAGE gel and transferred onto NC membrane (Millipore), followed by being incubated with the appropriate primary antibodies and second antibodies and then photographed by LI-COR or Tanon 4800 luminous imaging system. The primary antibodies are listed in Table 1.

### Realtime quantitative PCR

Total RNAs were extracted with Trizol (Sigma). 500 ng of total RNA was reverse-transcribed into cDNA with the Rever TraAce qPCR RT Kit (TOYOBO). Quantitative PCR was performed with SYBR Green Realtime PCR Master Mix (TOYOBO). All the experiments were performed according to the instructions and GAPDH was used as an internal control. All PCR primers are listed in Table 3.

**Table 3:** Primers for RT-PCR and mouse genotyping.

Gene	Forward sequence (5’–3’)	Reverse sequence (5’–3’)
mouse Ifnβ1	TCCGAGCAGAGATCTTCAGGAA	TGCAACCACCACTCATTCTGAG
mouse Cxcl10	GCCGTCATTTTCTGCCTCA	CGTCCTTGCGAGAGGGATC
mouse Isg15	TGACTGTGAGAGCAAGCAGC	CCCCAGCATCTTCACCTTTA
mouse Ifit1	GAACCCATTGGGGATGCACAACCT	CTTGTCCAGGTAGATCTGGGCTTCT
mouse Actin	GGCTGTATTCCCCTCCATCG	CCAGTTGGTAACAATGCCATGT
mouse Il6	GGATACCACTCCCAACAGACCT	GCCATTGCACAACTCTTTTCTC
mouse Ifna4	ACTGGTCAGCCTGTTCTCTAGGA	GGACTGTCAAGGCCCTCTTG
human IFIT1	GCGCTGGGTATGCGATCTC	CAGCCTGCCTTAGGGGAAG
human IFI27	TGCTCTCACCTCATCAGCAGT	CACAACTCCTCCAATCACAACT
human SIGLEC1	ATGGGGTACGCCTCCAAAC	GTGCCTCATTGGGTGTGTTG
human IFI44L	ACAGAGCCAAATGATTCCCTATG	TCGATAAACGACACACCAGTTG
human ACTIN	ACACTGTGCCCATCTACGAG	TCAACGTCACACTTCATGATG
human GAPDH	GAGTCAACGGATTTGGTCGT	TTGATTTTGGAGGGATCTCG
Cgas K350R genotyping	AAATGGATCAACCCTTTCTTTGC	TGCTGTGGCTTGGAAGTTATTCT
Cgas K350M genotyping	AAATGGATCAACCCTTTCTTTGC	TGCTGTGGCTTGGAAGTTATTCT
Mouse Kdm4b genotyping	F1:TAGGGTTGGAAGGGGACCTGATG	R1:GCAGCCCAGGTTATTGTTTCCTG
	F2:GGTTGGCTATGGGCTCTGTCTCT	R2:ACTGAACTGGAGGCCAGAGAATG
Mouse Trex1 genotyping	AGGCAAATAAGTAGTGGATC	TCTCACTGGCCCCAGGGCTAC
Human TREX1 genotyping	CTGAGATGTGCTTCTGCCCA	ATCCTTGGTACCCCTGCTCT

### Mouse genotyping

500 μl mouse lysate (10 mM Tris–Cl [pH 8.0], 5 mM EDTA, 200 mM NaCl, 0.2% SDS) and 5 μl proteinase K were added for digestion of mouse toes and lysed overnight at 55 °C. Then 700 μl phenol–chloroform was added, and the supernatant was mixed with isopropanol. After centrifugation and washing with pre-cooled 70% ethanol, the precipitate was added with 50 μl ddH_2_O. PCR experiments were performed with designed primers, followed by DNA electrophoresis. Mouse genotypes were determined by DNA size. All PCR primers for genotyping are listed in Table 3.

### Subcellular fractionation

The cells were rinsed with ice-cold PBS and then added buffer A (10 mM HEPES [pH 7.9], 10 mM KCl, 1.5 mM MgCl_2_, 0.5 mM DTT, 0.05% NP40, and protease inhibitor cocktail). After 15 min on ice, the cells were centrifuged at 3000 rpm and 4 °C for 10 min. The resultant supernatant was used as a cytosolic fraction (Cyto.). The pellet was then homogenized with buffer B (20 mM HEPES [pH 7.9], 0.4 M NaCl, 1 mM EDTA, 1 mM DTT, and 1 mM PMSF). After 10 min of vigorous vortex, the homogenates were centrifuged at 13,000 rpm and 4 °C for 20 min. The resultant supernatants were used as soluble nuclear fractions (N1). The pellets were then used as insoluble nuclear fractions (N2). Each fraction was analyzed by SDS–PAGE. GAPDH, CREB, and H3 are markers for cytoplasm, soluble and insoluble nuclear fractions, respectively.

### In vivo tumor growth and treatments

For the in vivo tumor growth experiments, 6–8 weeks C57BL/6 mice were randomly divided into four groups and inoculated subcutaneously with 1 × 10^6^ shKDM4B and/or sgcGAS B16F10 cells in 100 μl saline as indicated. Anti-mouse PD-1 combined with DMSO (10%) in PBS were given intraperitoneally at a dose of 200 μg per mouse on day 7 after tumor cell inoculation, The tumor volumes were measured along two orthogonal axes (length and width) and were calculated as follows: tumor volume = length × width × width/2. In none of the experiments did tumor size surpass 2 cm in any dimension. For combination treatments, mice were planted subcutaneously with 1 × 10^6^ sgKDM4B or sgNC B16F10 cells in 100 μl saline. Mice were randomly divided into two groups. Anti-mouse PD-1 or rat IgG2a isotype combined with DMSO (10%) in PBS were given intraperitoneally at a dose of 200 μg per mouse on day 7 after tumor cell inoculation, then every 3 days for the duration of the experiments. For the HSV-1 treatment, *Kdm4b* KO and WT mice were injected intraperitoneally at a dose of 1 × 10^6^ pfu per mouse and harvested the lungs, spleen and heart at the indicated time. The survivors were monitored for 8 days. For JIB-04 treatment, *Trex1*^*−/−*^ and WT mice were injected intraperitoneally at the concentration of 20 mg/kg daily and harvested the spleen and heart at the indicated time. The survivals were monitored for over 3 months. All the drug studies employed a double-blinded design, and animals were monitored daily for clinical signs of toxicity, including weight loss, lethargy, ruffled fur, or abnormal behavior.

### Immunohistochemistry

The tissue specimens were fixed overnight in 10% neutral-buffered formalin and then were dehydrated in increasing concentrations of isopropyl alcohol, followed by clearing of alcohol by xylene. The specimens were subsequently embedded in paraffin wax in cassettes for the facilitation of tissue sectioning. Standard staining with hematoxylin and eosin (H&E) was performed on Section 3 μm thickness from each specimen block.

### Quantification and statistical analysis

GraphPad Prism5 software was used for data analysis. Data were shown as mean ± SEM from at least three independent experiments (*n* ≥ 3). Statistical significance was determined by *t*-test (two-tailed) for two groups unless otherwise indicated. *P* value 0.05 was considered statistically significant. In the graphed data, *P < 0.05, **P < 0.01, ***P < 0.001, ****P < 0.0001, ns, not significant.

## Supplementary information

Supplemental Figures

WB and QPCR original data

## Data Availability

The datasets generated and analyzed in the current study are available from the corresponding author on reasonable request.
