# Supplementary material for: KDM4B enhances immune surveillance via demethylating cGAS
Source: Cell Death Dis. 2025 Jul 1;16(1):478. doi: 10.1038/s41419-025-07792-w (PMC12215012; doi:10.1038/s41419-025-07792-w)
Supplement: Supplementary file 1 — Supplemental Figures [file 41419_2025_7792_MOESM1_ESM.docx]

**KDM4B Enhances Immune Surveillance via Demethylating cGAS**

*Qiao Peng, Huimin Zhuo, Minkang Wu, Yun Hao, Yiyi Zhang, Yuying Zheng, Lei Yu, Lin Han, Hui Ren, Yingcong Wang, Zhijie Gao, Leilei Wu, Qi Lin, Chunhua Lu, Jinghua Li, Ping Wang, Lan Fang, Haihong Yu and Meiling Lu.*

**Fig. S1**


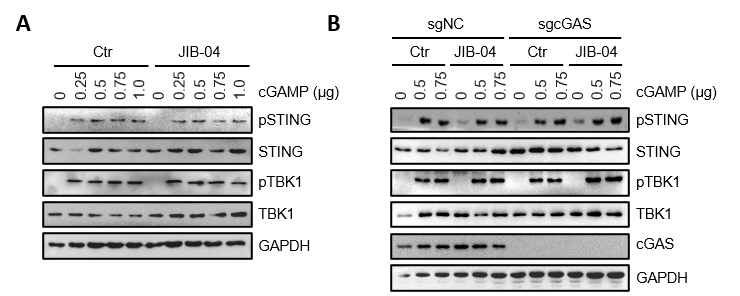


**Fig. S1 JIB-04 did not suppress cGAS-STING activation induced by cGAMP treatment.**

**A)** Effects of JIB-04 treatment on cGAS-STING activity in B16F10 with or without cGAMP stimulation. **B)** Effects of JIB-04 treatment on cGAS-STING activity in B16F10 *Cgas* KO and control cells with or without cGAMP stimulation. Different concentration of cGAMP were transfected into B16F10 cells respectively, 24 hr later, cells were treated with 20 μM JIB-04 for 12 hr.

**Fig. S2**


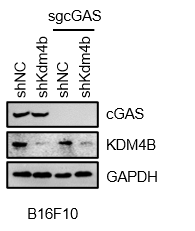


**Fig. S2 *Cgas* and *Kdm4b* double knockout cell lines generated in B16F10.**

**Fig. S3**


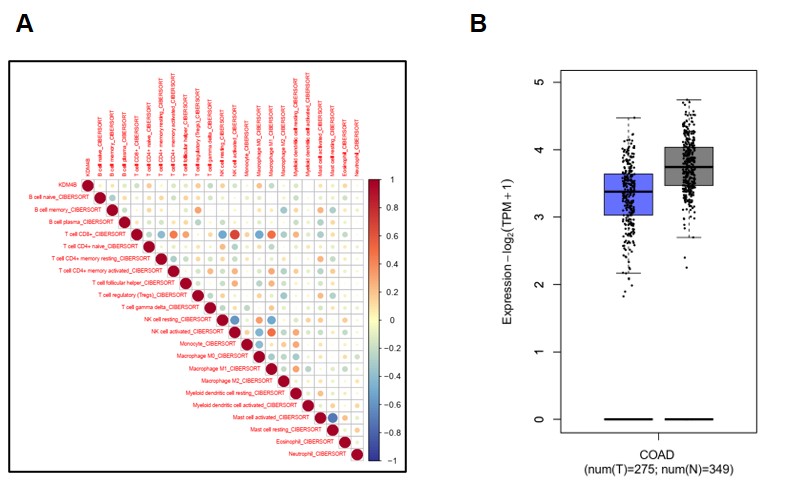


**Fig. S3 *TCGA analysis of Kdm4b* expression in colorectal cancer (COAD) and immune cell infiltration in melanoma.**

**A)** The correlation of *Kdm4b* expression with immune cell infiltration in melanoma microenvironment. Applying the CIBERSORT algorithm on TCGA dataset, we performed Spearman correlation analysis between KDM4B expression and the abundance of 22 immune cell types in melanoma. The color of the circle represents the negative correlation and the size of the circle represents the significance. **B)** TCGA analysis for *Kdm4b* mRNA expression in COAD cells on the GEPIA website

**Fig. S4**


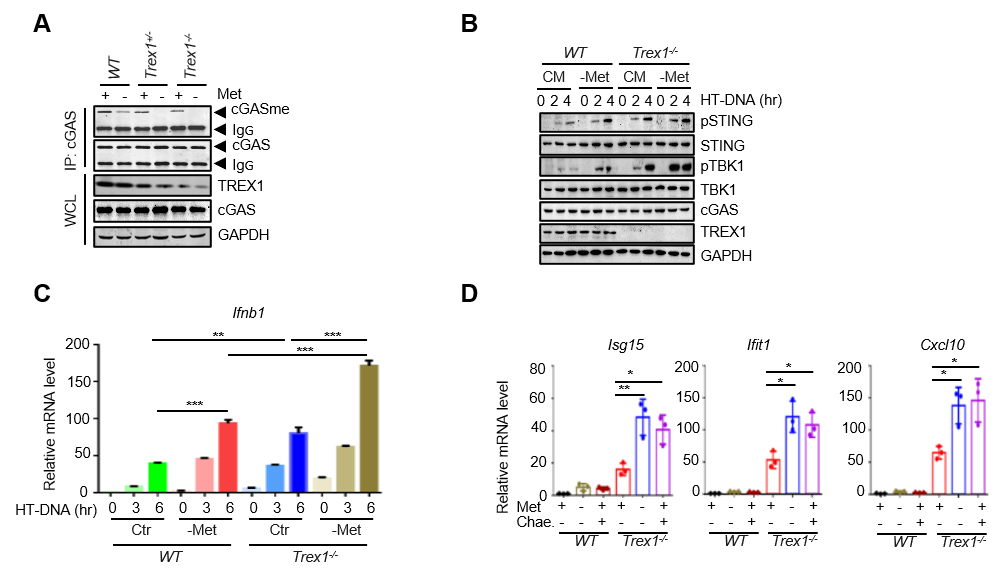


**Fig. S4 Methionine starvation decreases cGAS methylation and promotes *Ifnb1* signaling in autoimmune disease model**

**A-C)** Effects of methionine starvation on cGAS methylation (A), cGAS-STING activity (B), and *Ifnb1* expression (C) in bone marrow cells from *Trex1^-/-^* and wild-type (*WT*) mice. **D**) ISGs expression in bone marrow cells from *Trex1^-/-^* and wild-type (*WT*) mice treated with chaetocin (20 μM) and methionine starvation treatment for 12 hr. Data are mean ± SEM for C and D, one-way ANOVA.
