# Supplementary material for: KDM4B enhances immune surveillance via demethylating cGAS
Source: Cell Death Dis. 2025 Jul 1;16(1):478. doi: 10.1038/s41419-025-07792-w (PMC12215012; doi:10.1038/s41419-025-07792-w)
Supplement: Supplementary file 2 — WB and QPCR original data [file 41419_2025_7792_MOESM2_ESM.docx]

WB original data

Figure 1

A.


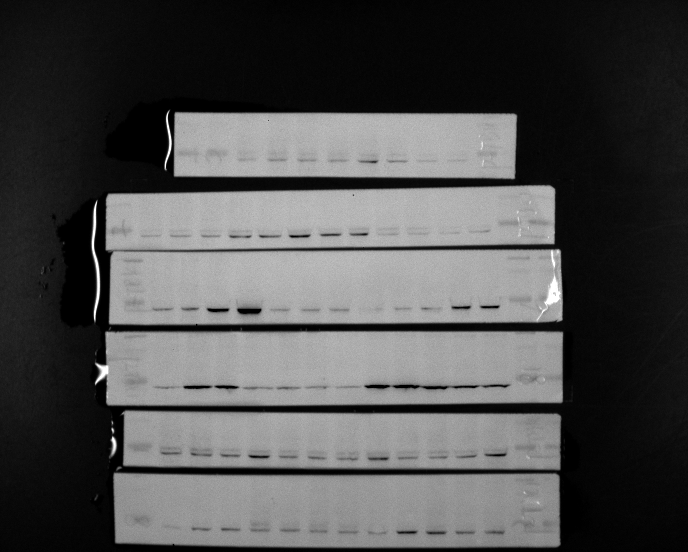

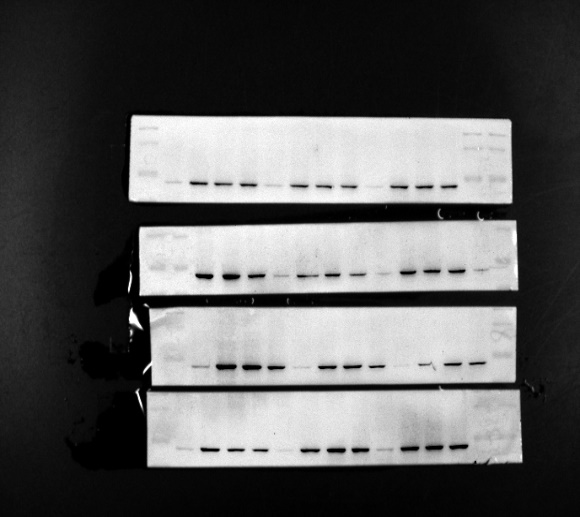

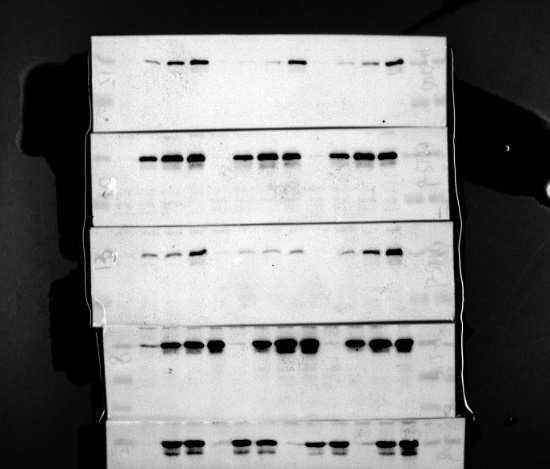

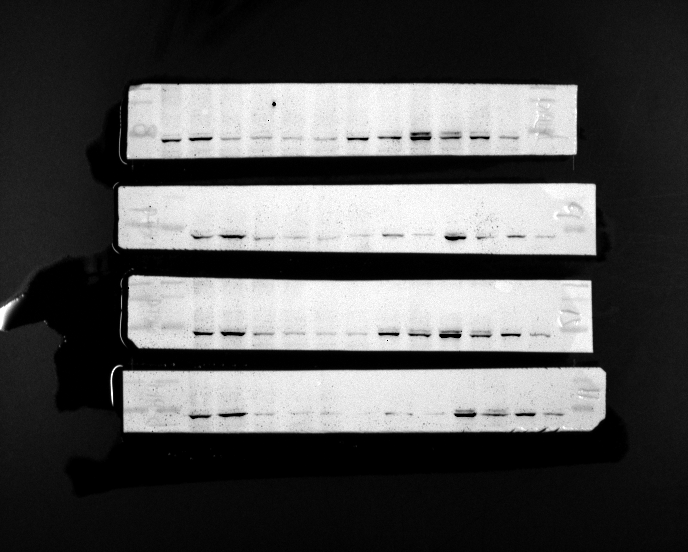

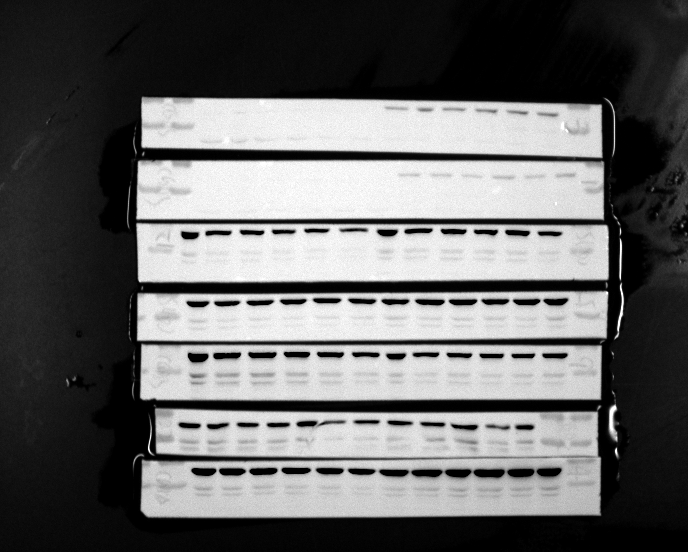

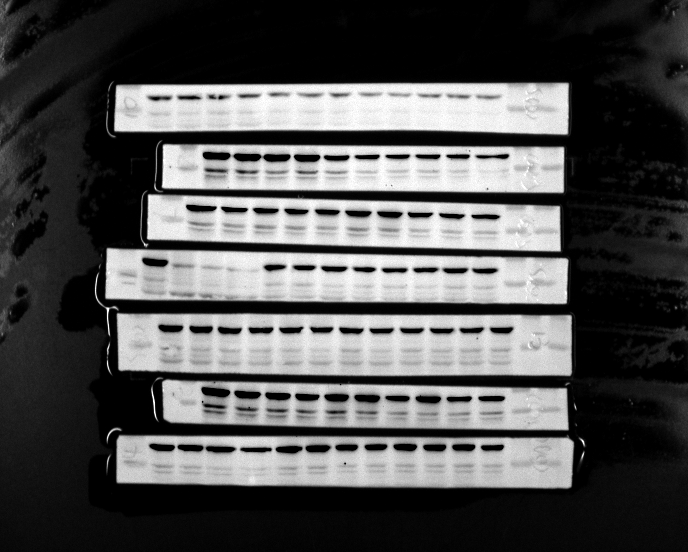

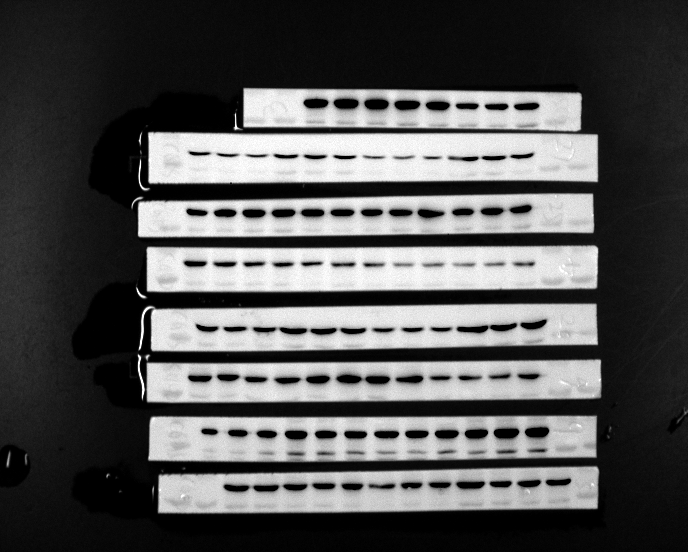

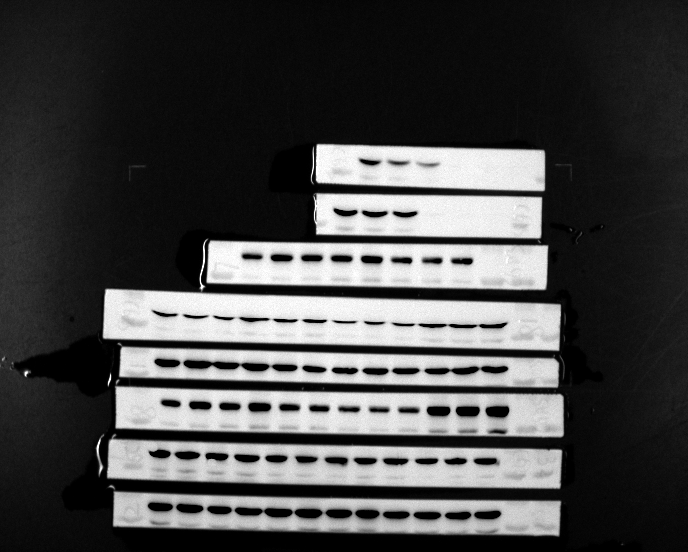

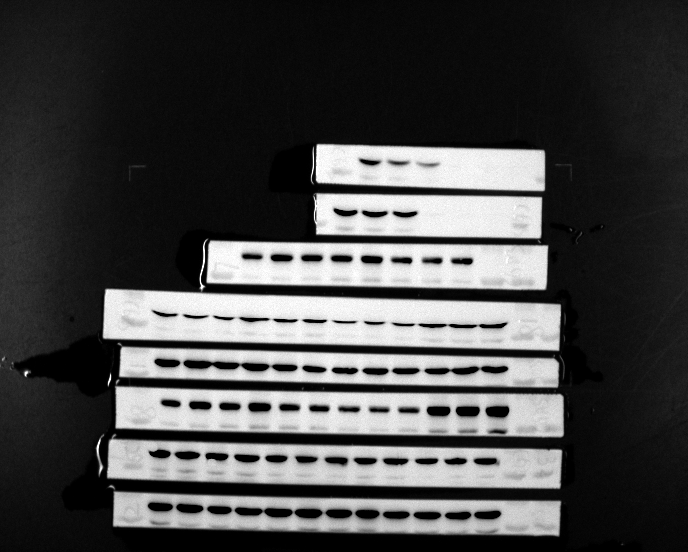

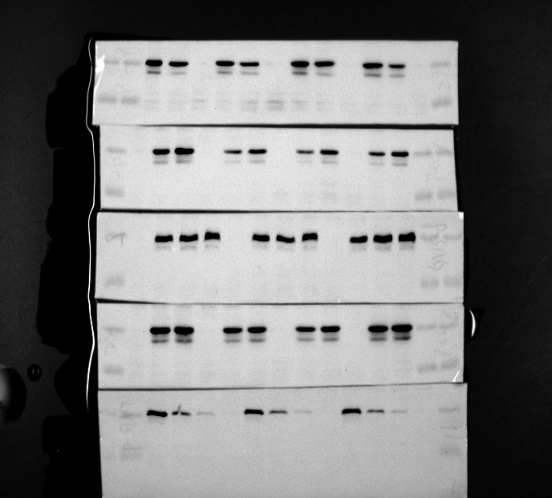

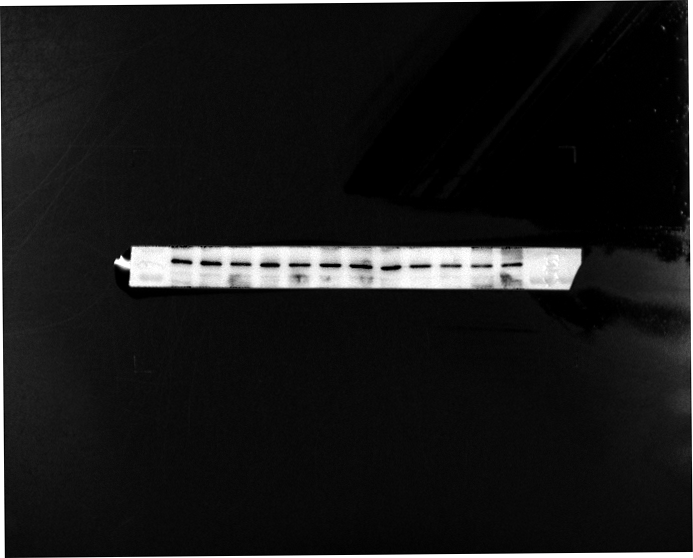

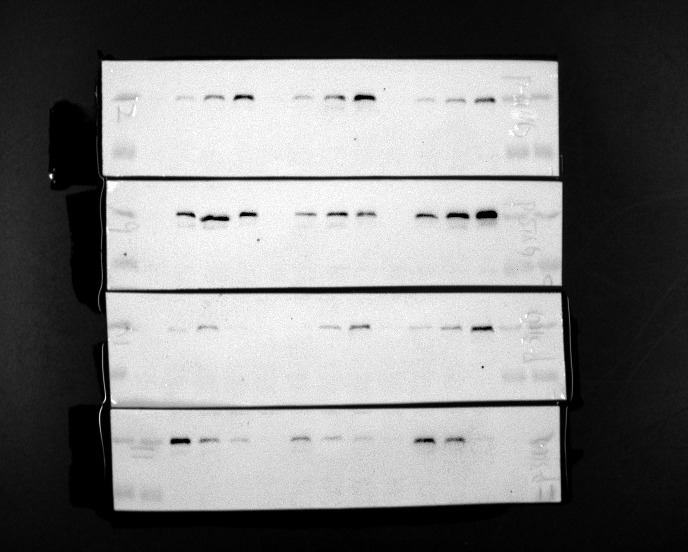

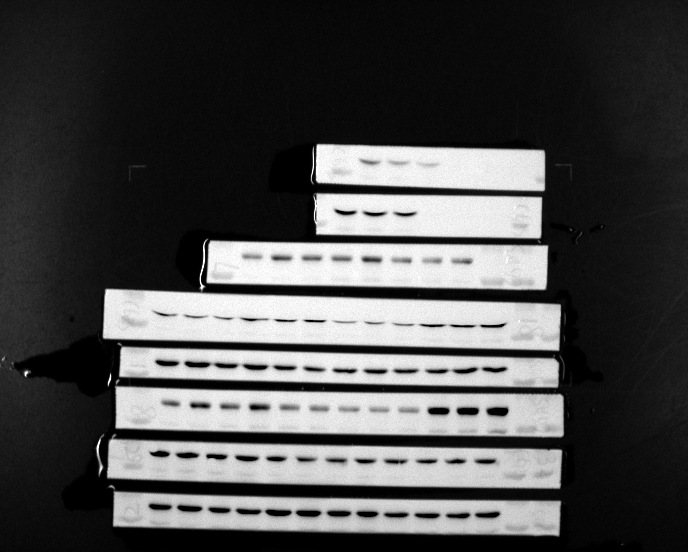

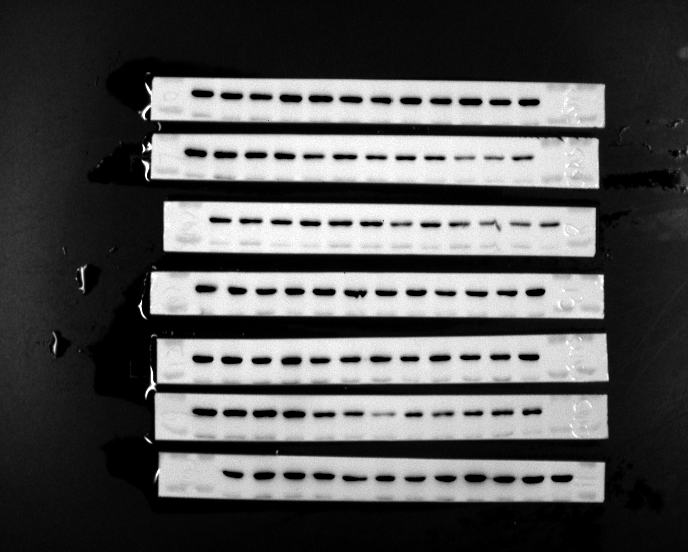

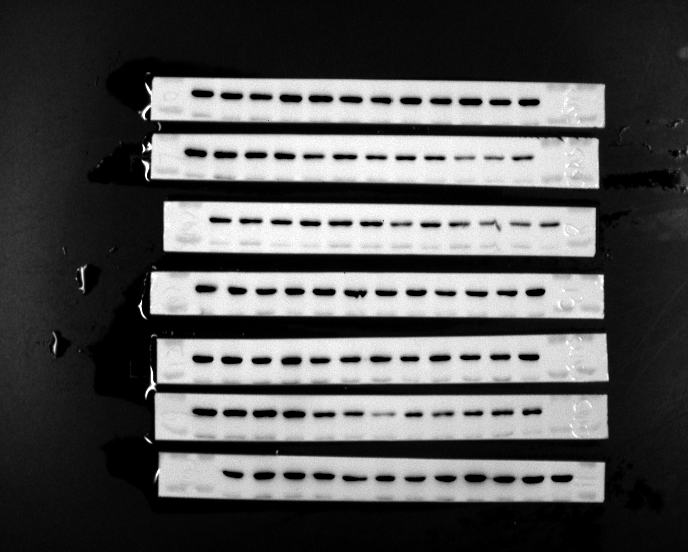

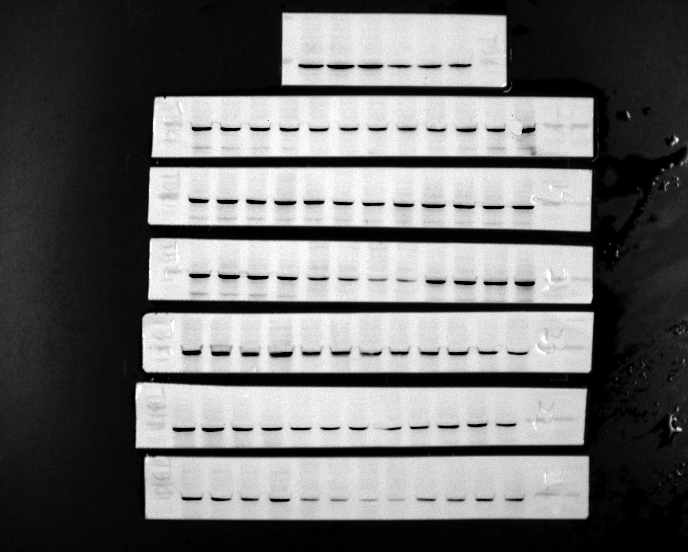

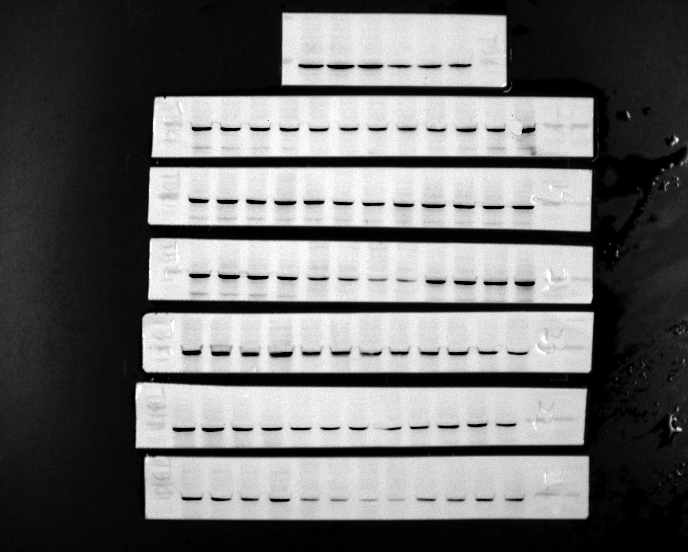

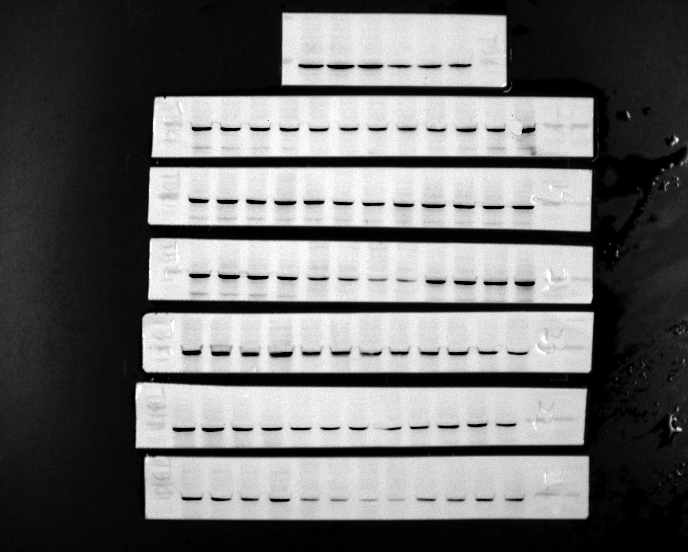


STING

TBK1

cGAS

GAPDH

pSTING

pTBK1

B.

pSTING


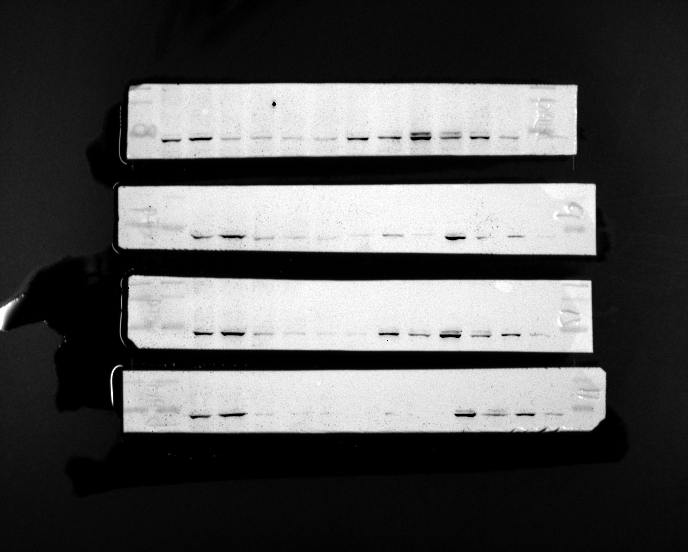

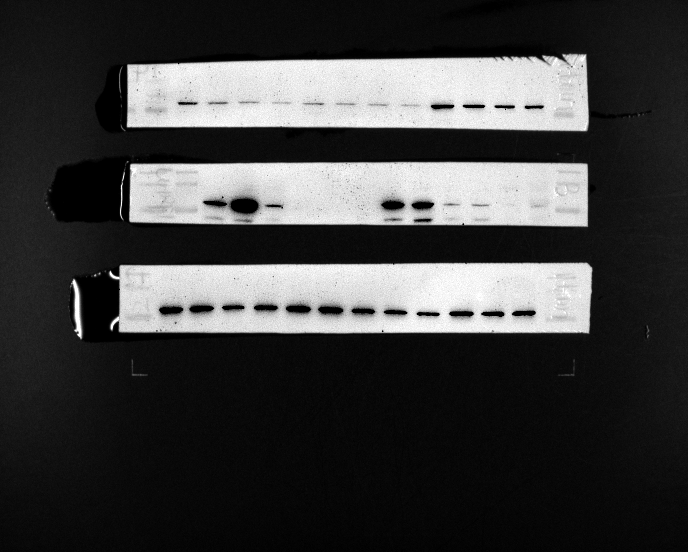

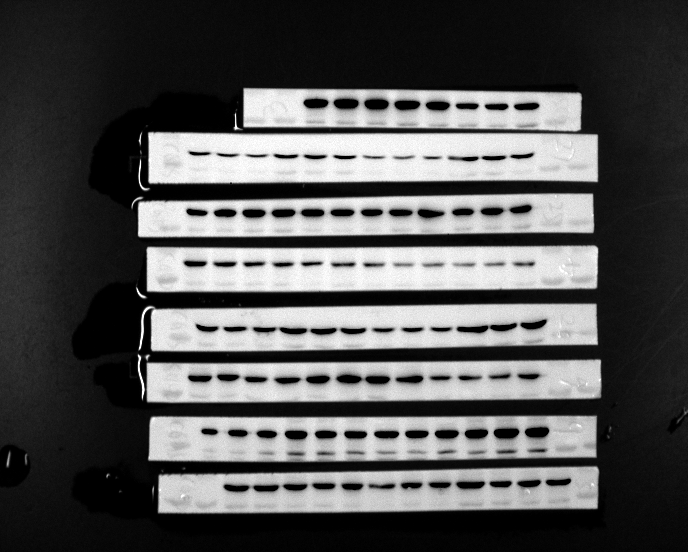

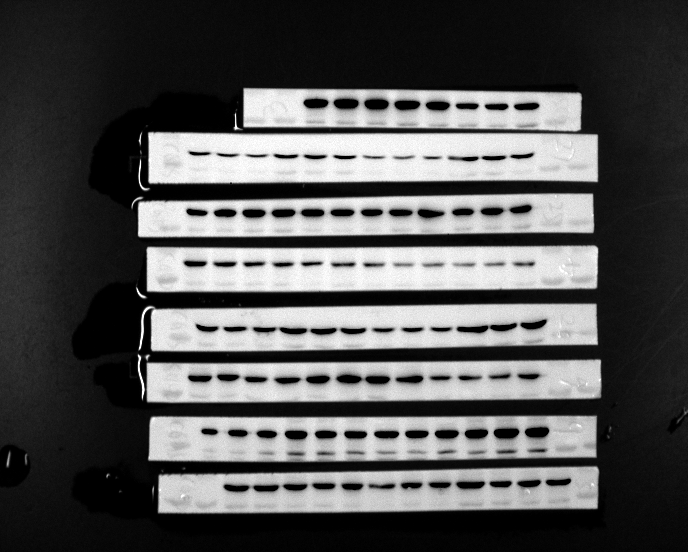

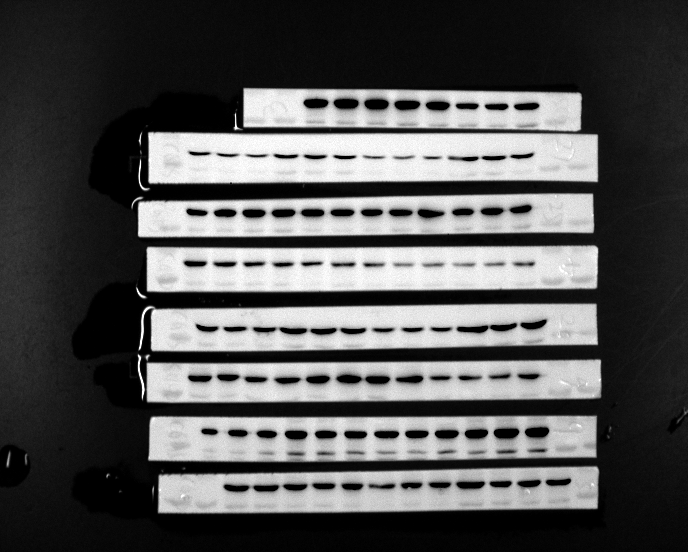

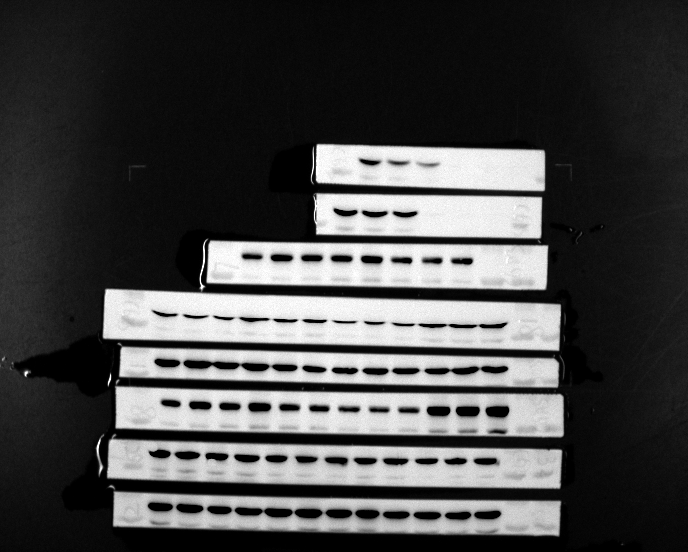

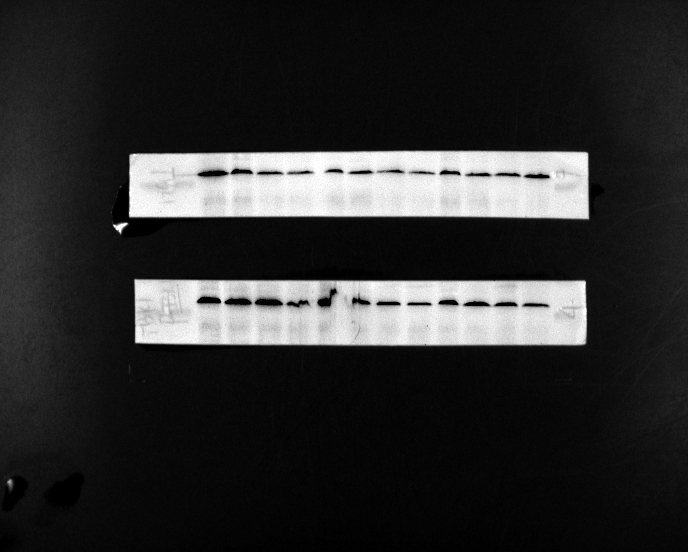

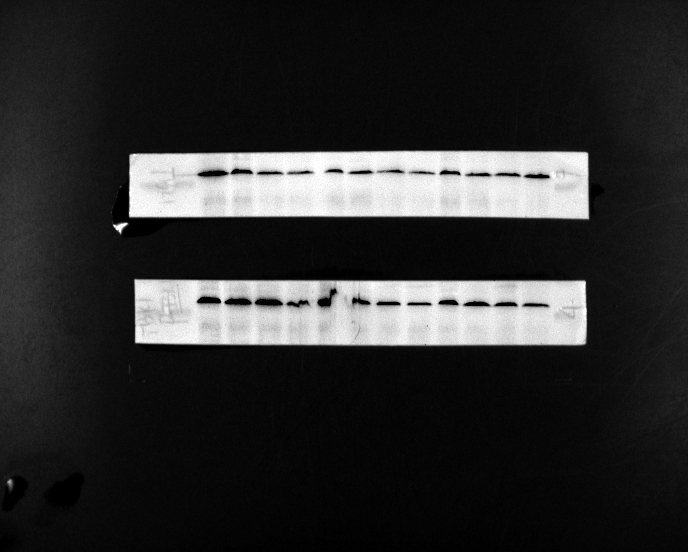

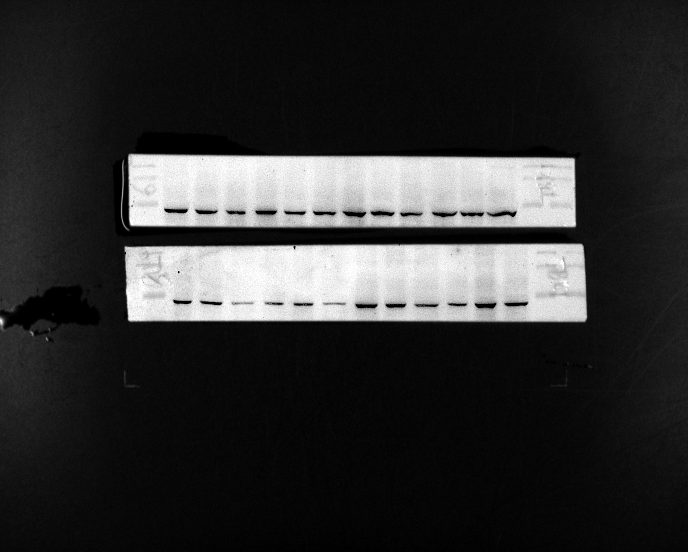

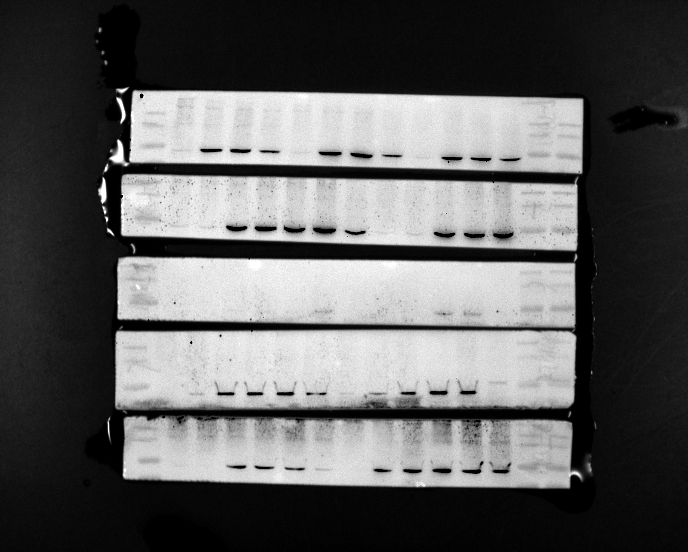

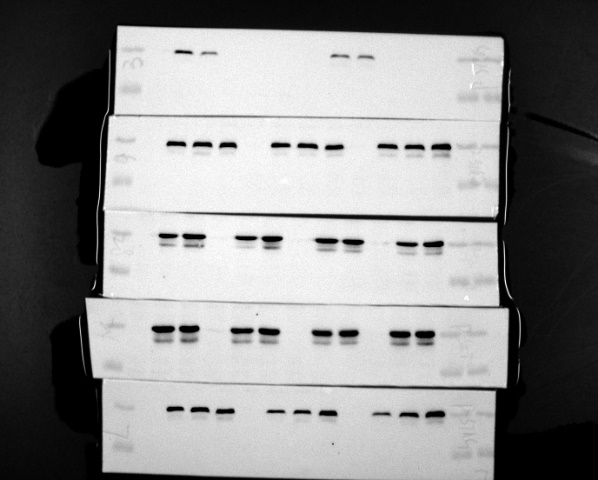

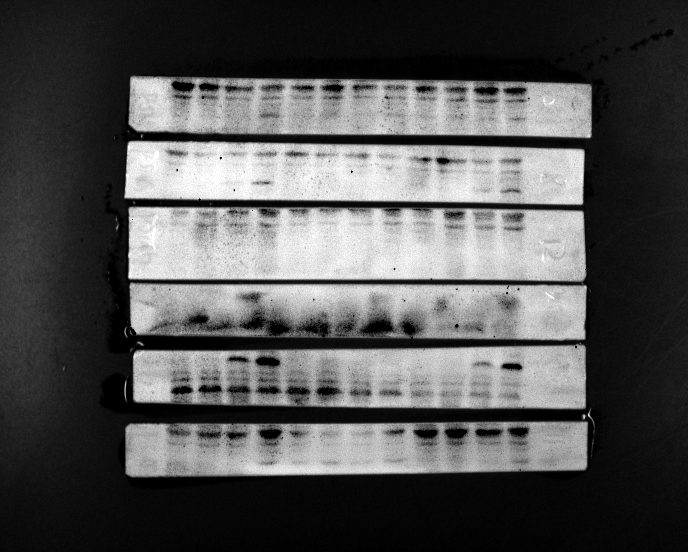

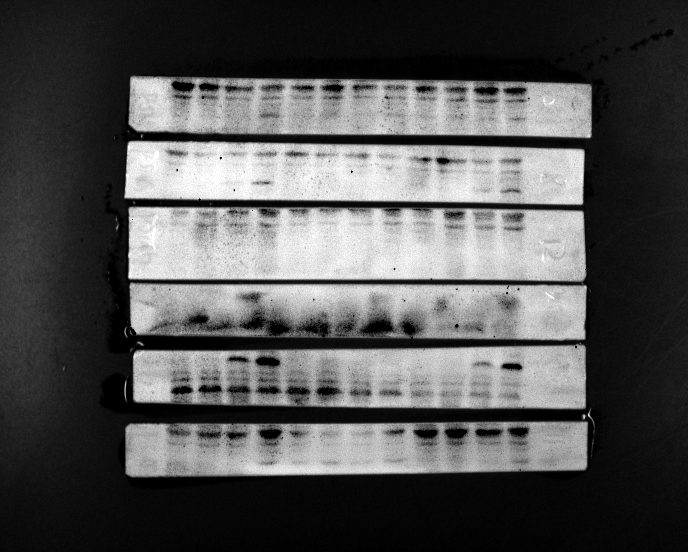

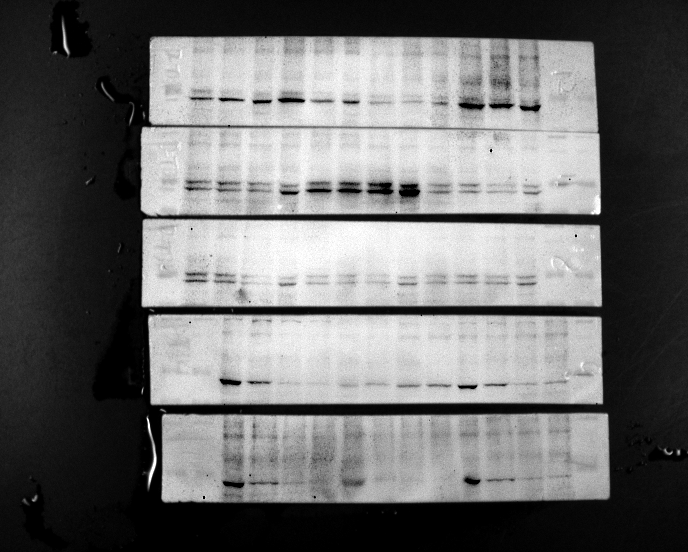

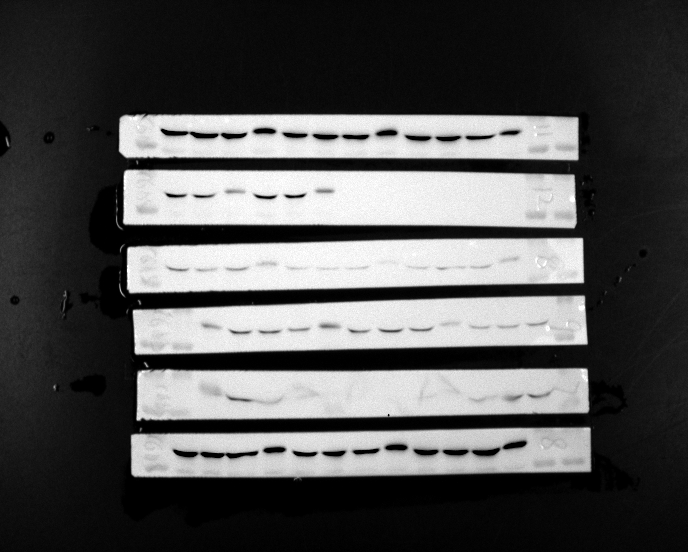

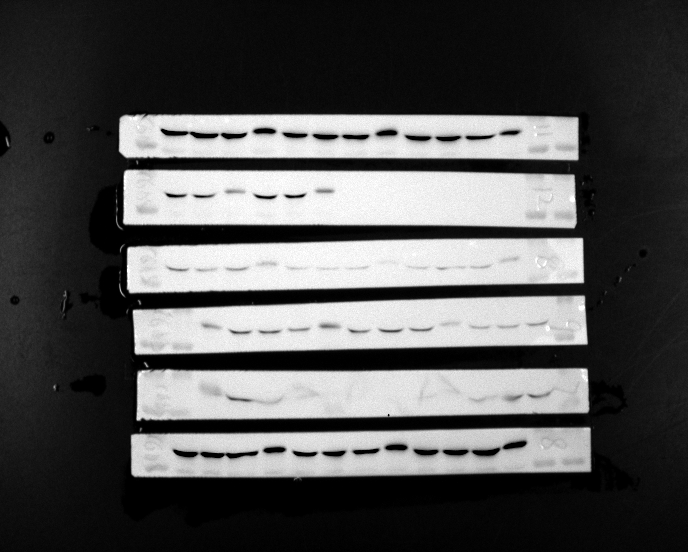

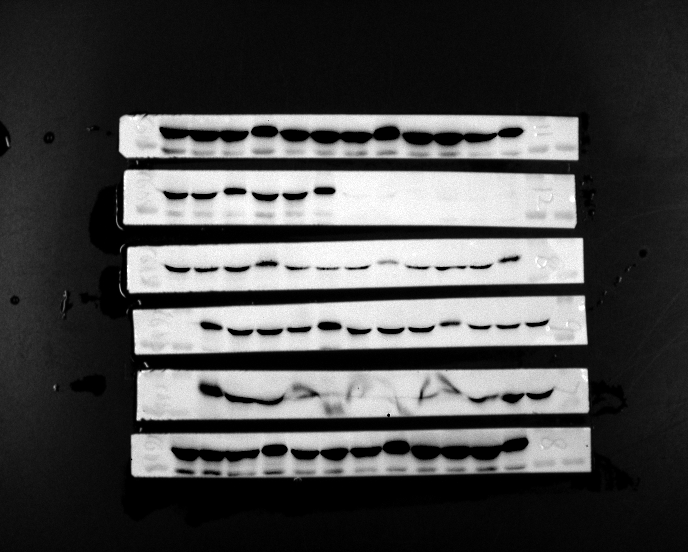


STING

pTBK1

TBK1

cGAS

GAPDH

D.


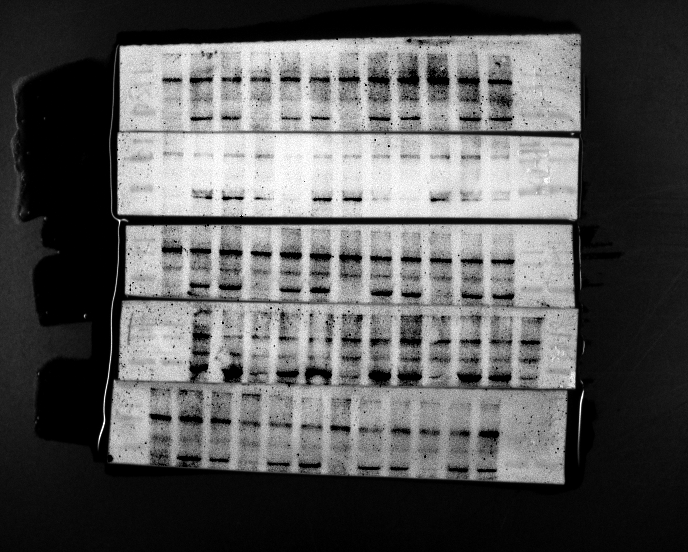

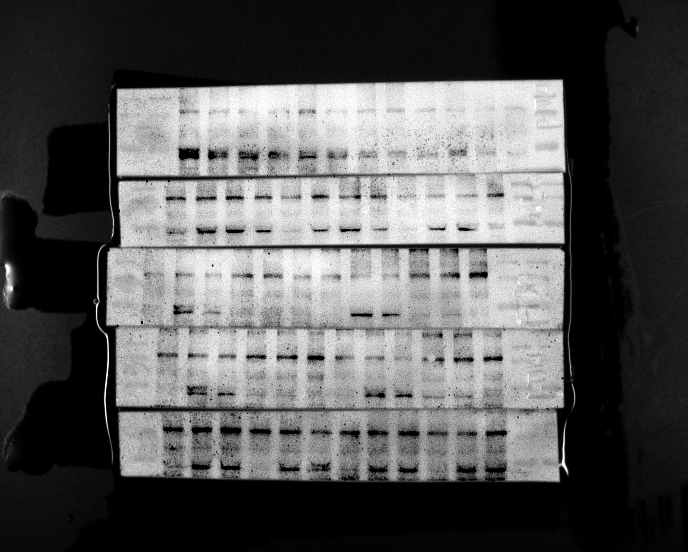

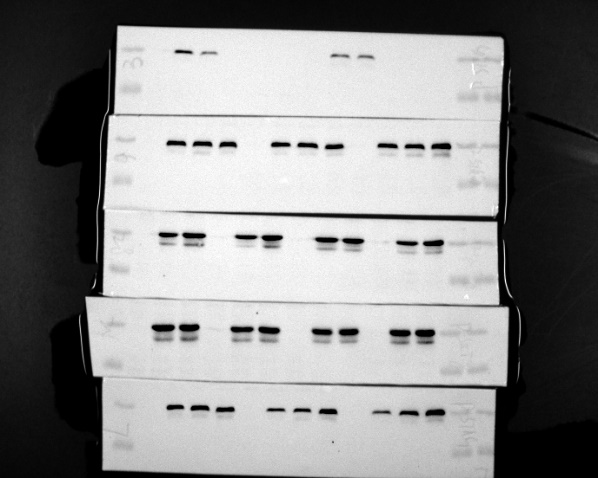

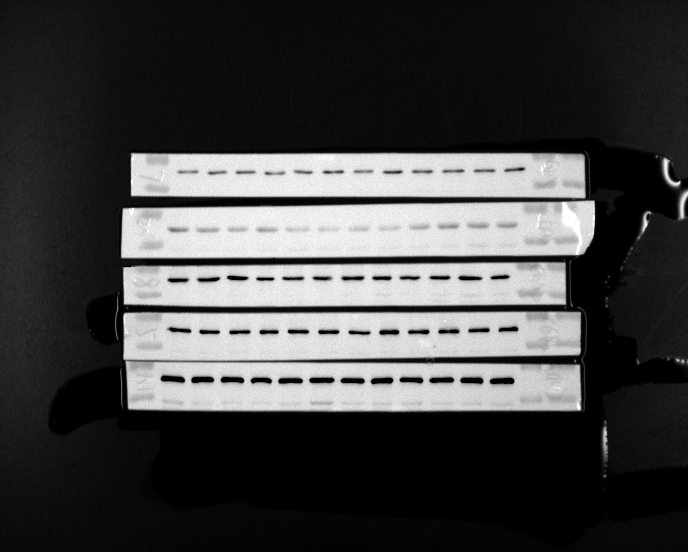

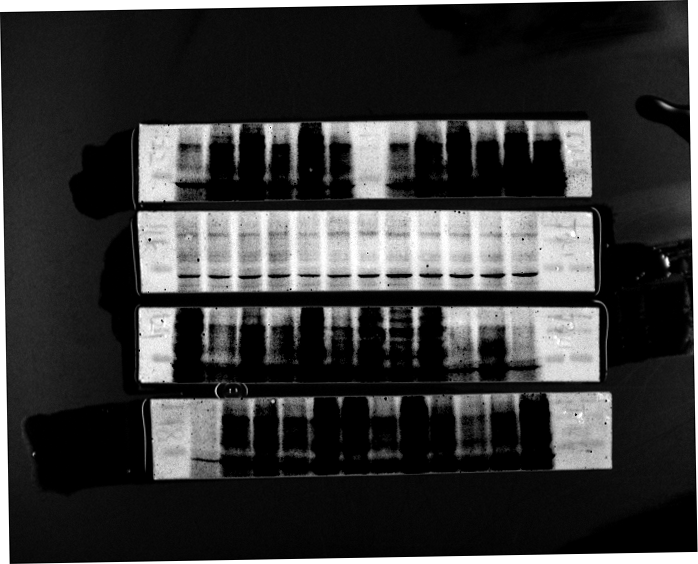

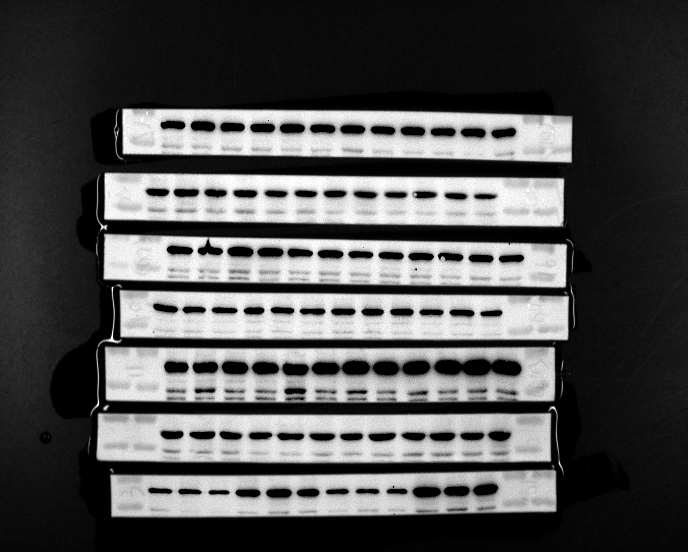

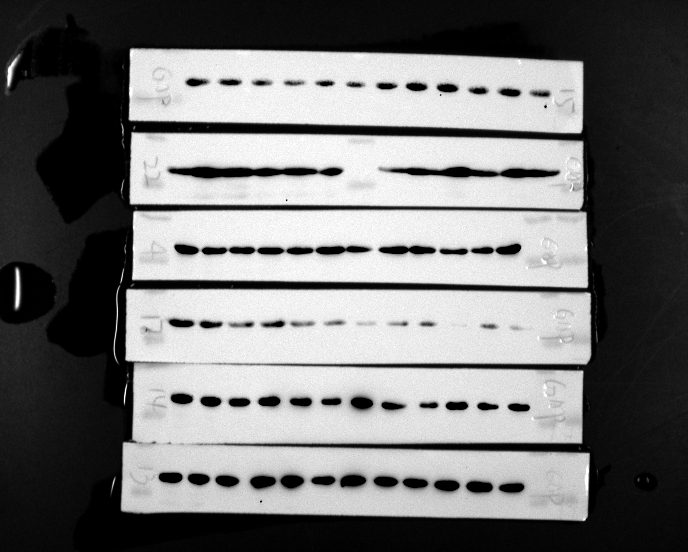

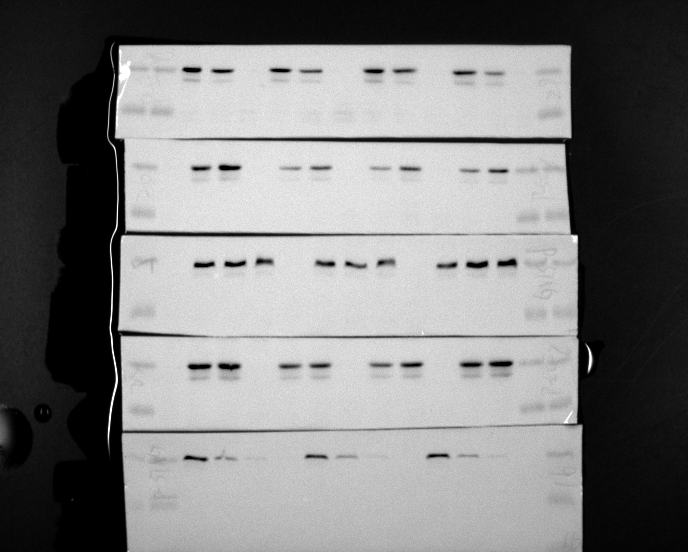

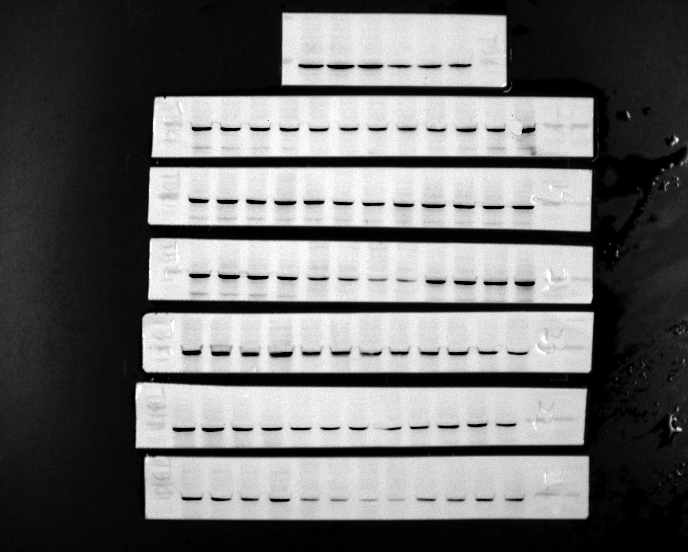

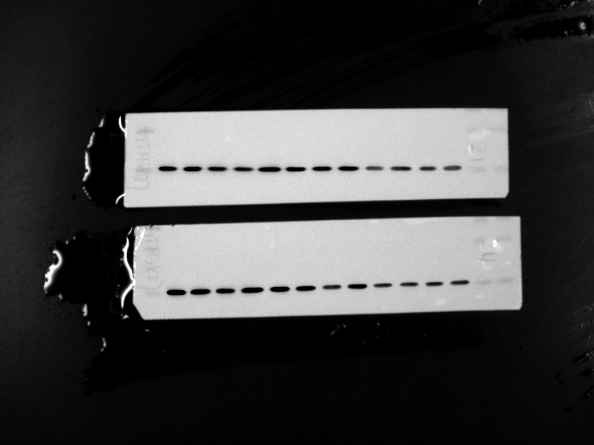

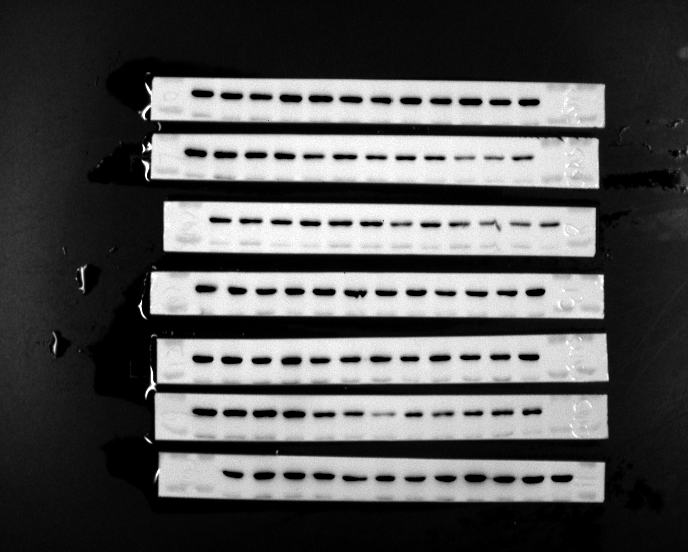

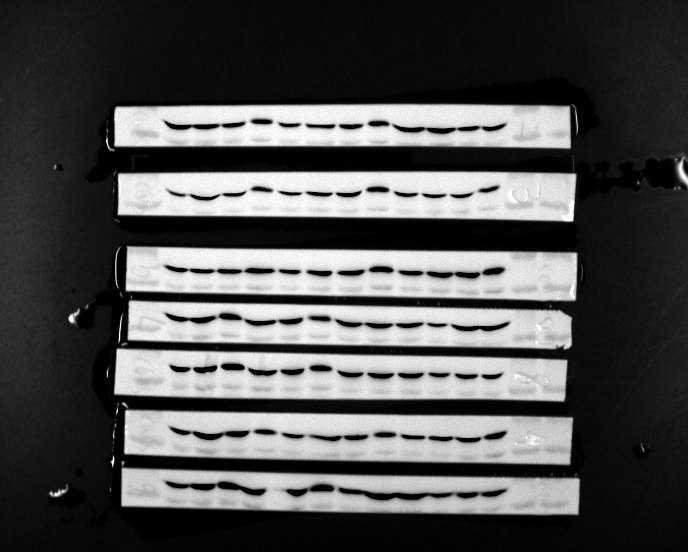


STING

pTBK1

TBK1

cGAS

GAPDH

pSTING

E.


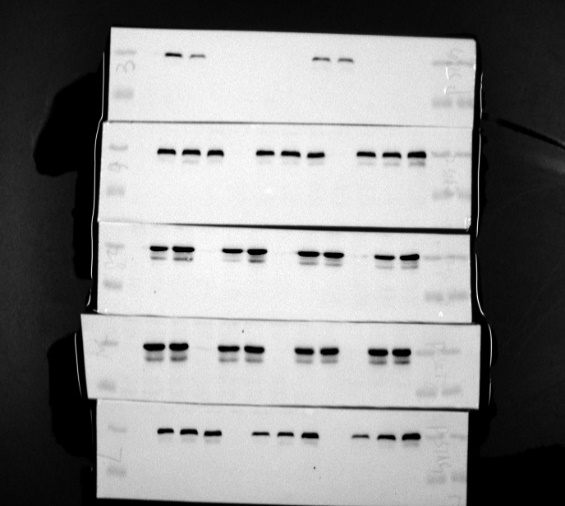

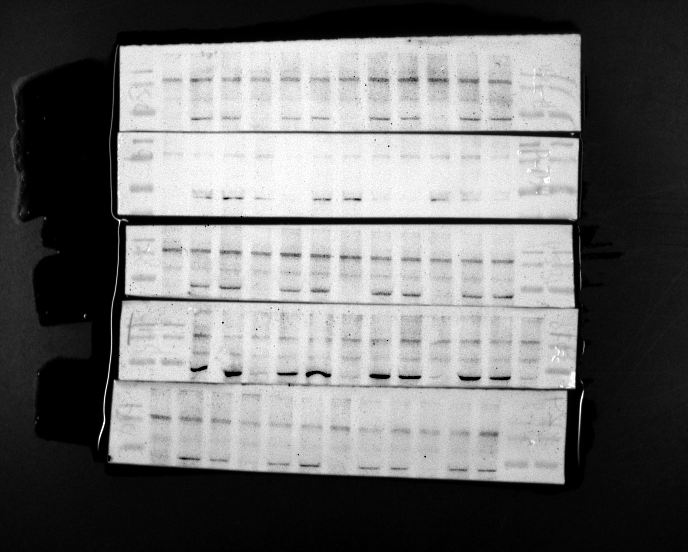

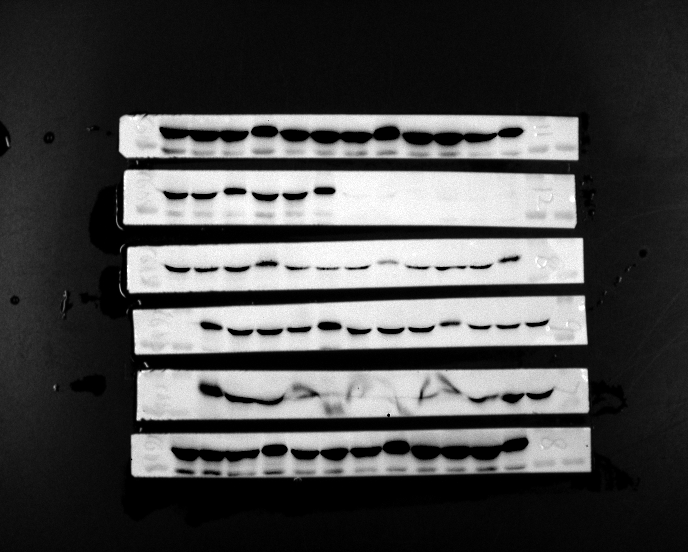

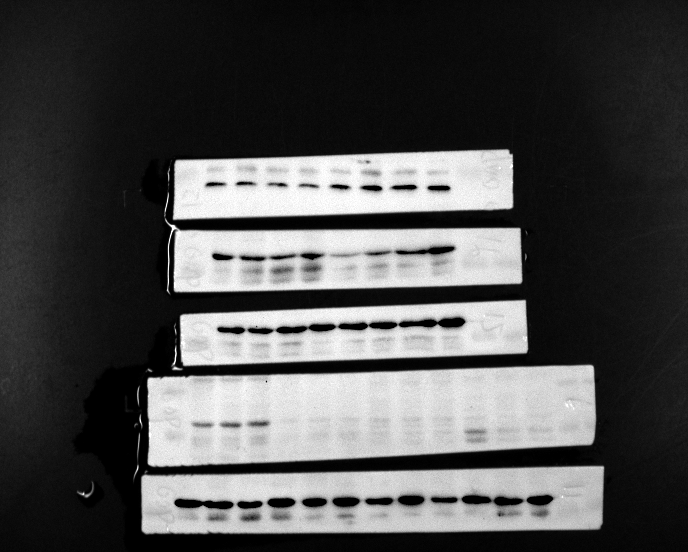

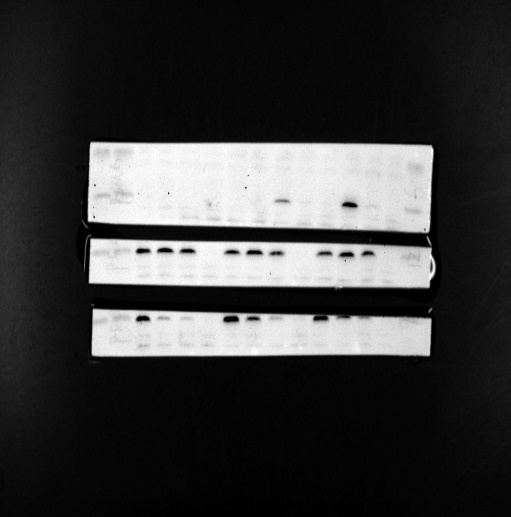

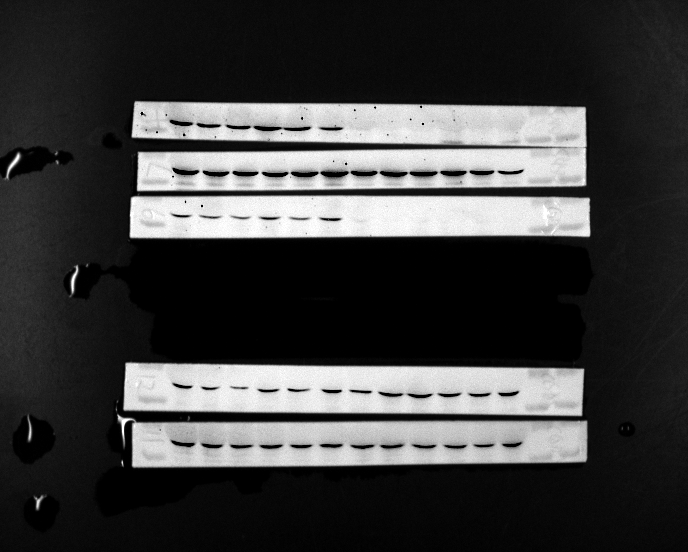

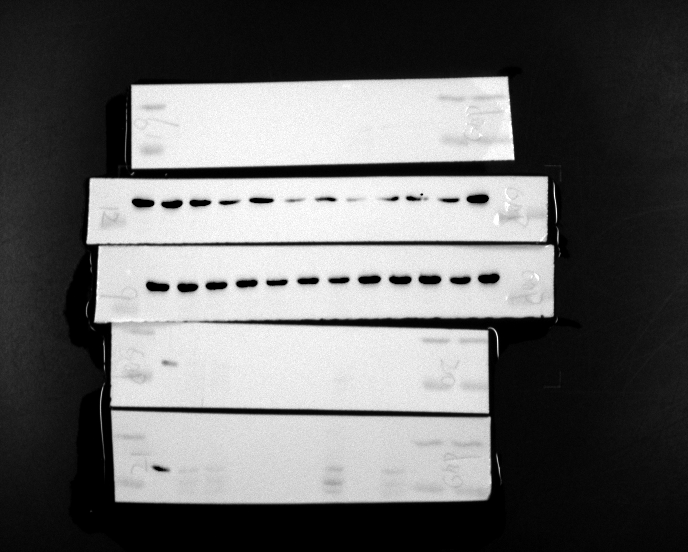

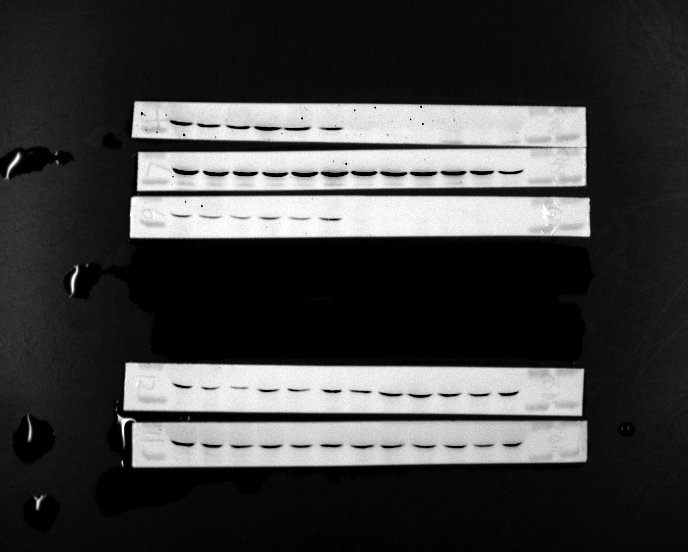

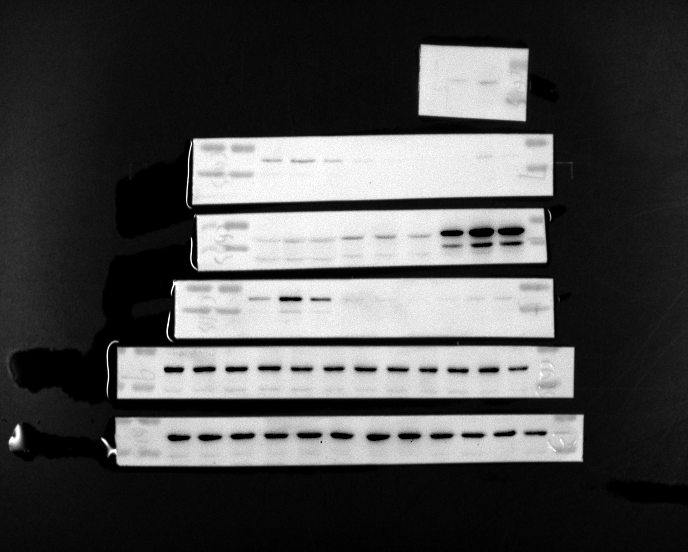

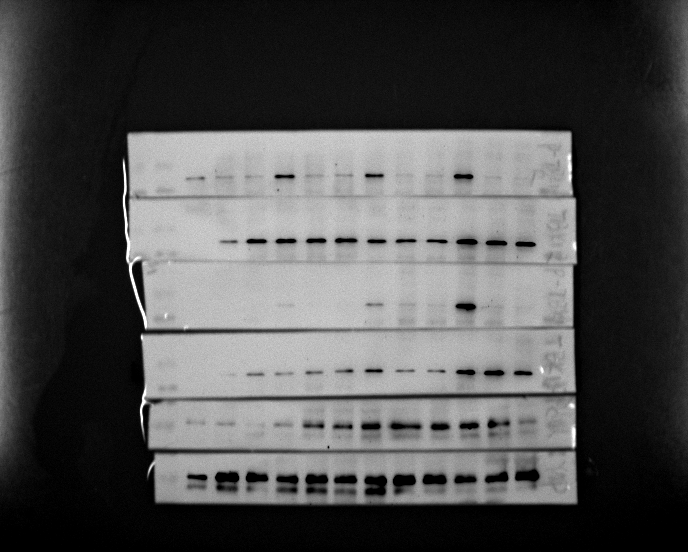

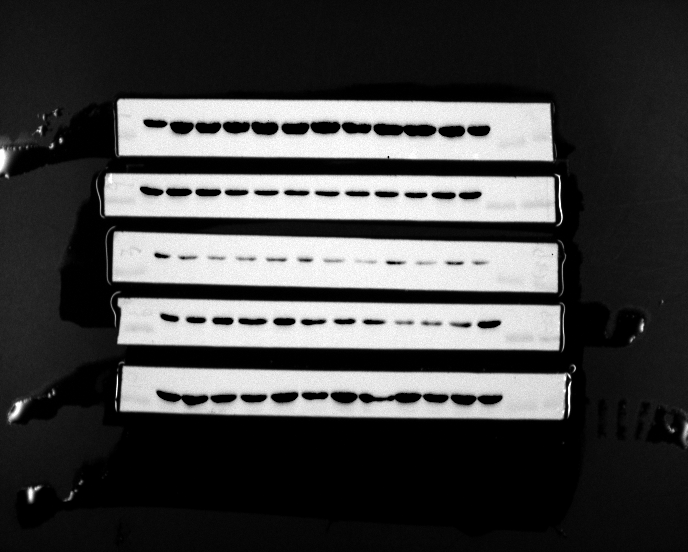

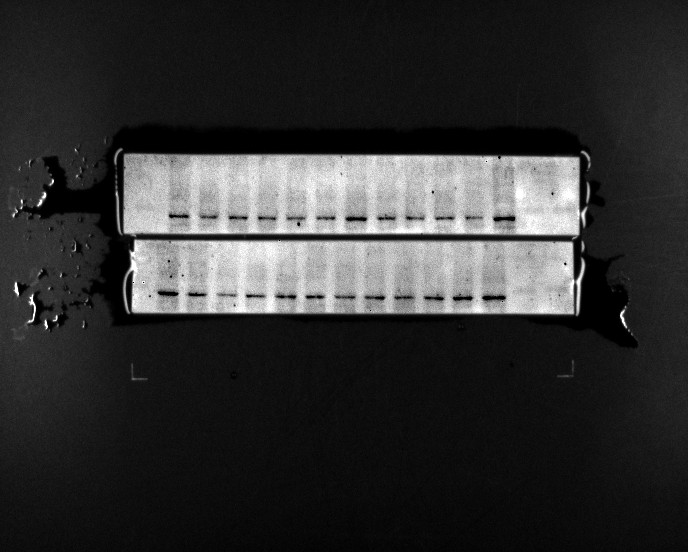


pSTING

STING

pTBK1

TBK1

cGAS

GAPDH

F.


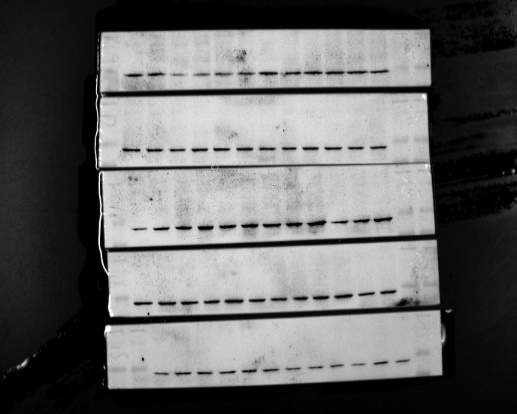

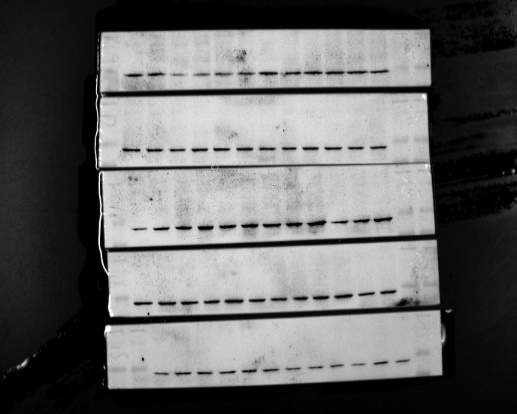

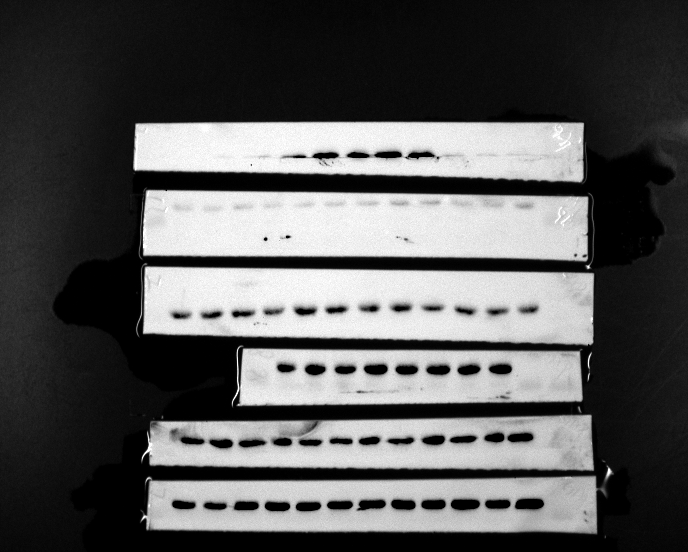

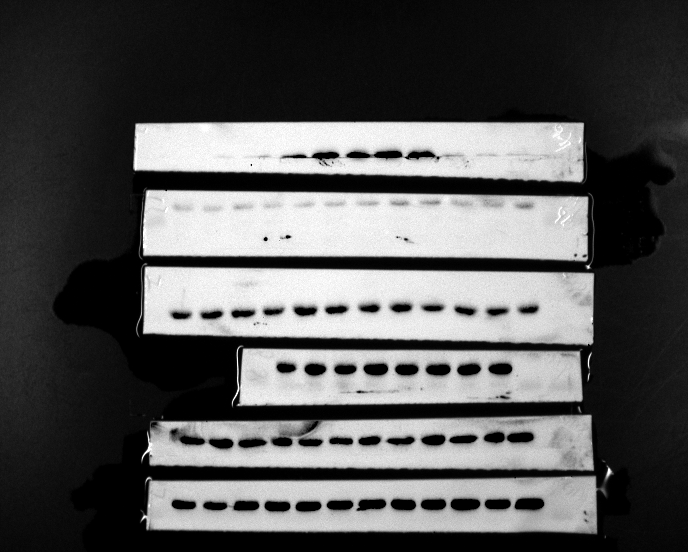

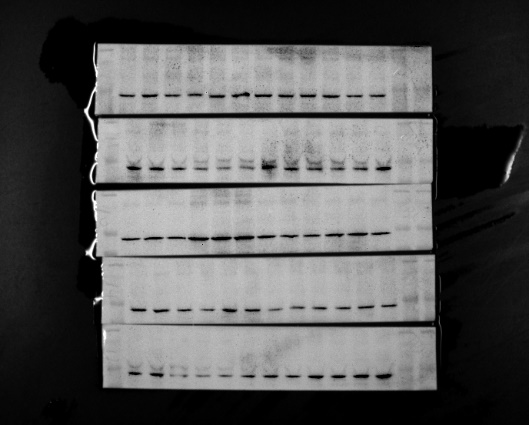

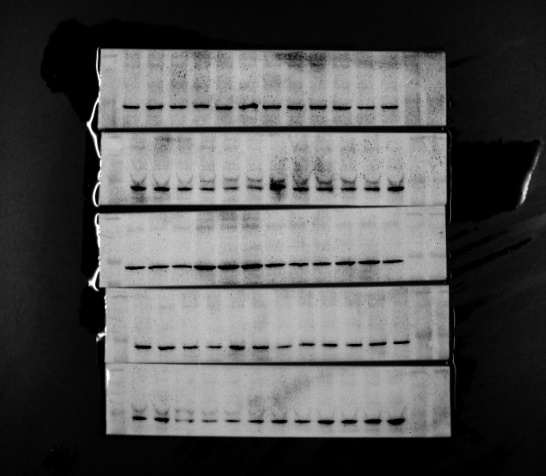

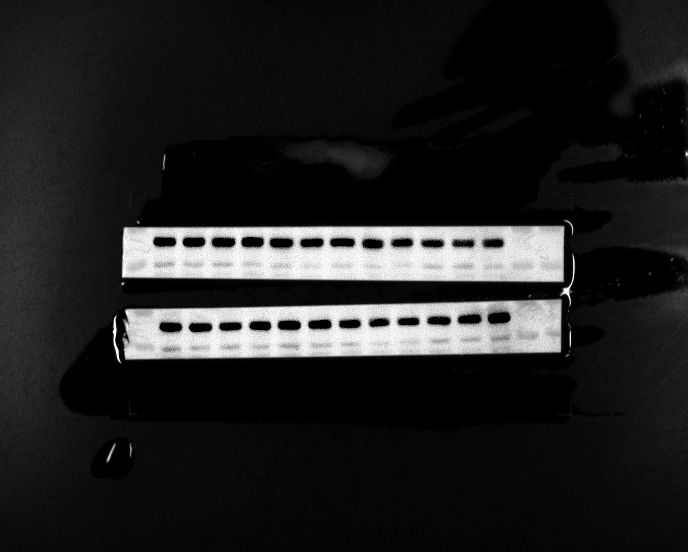

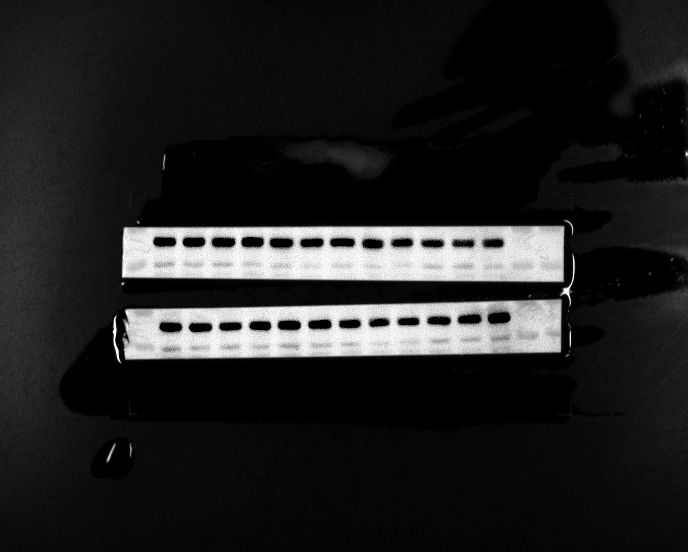

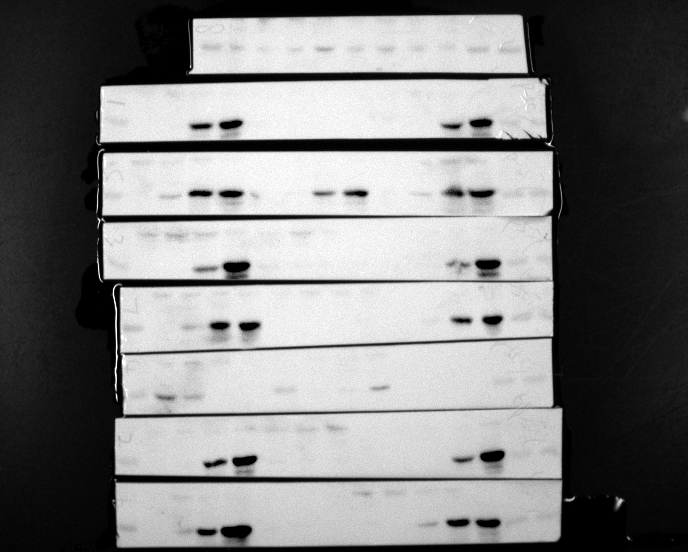

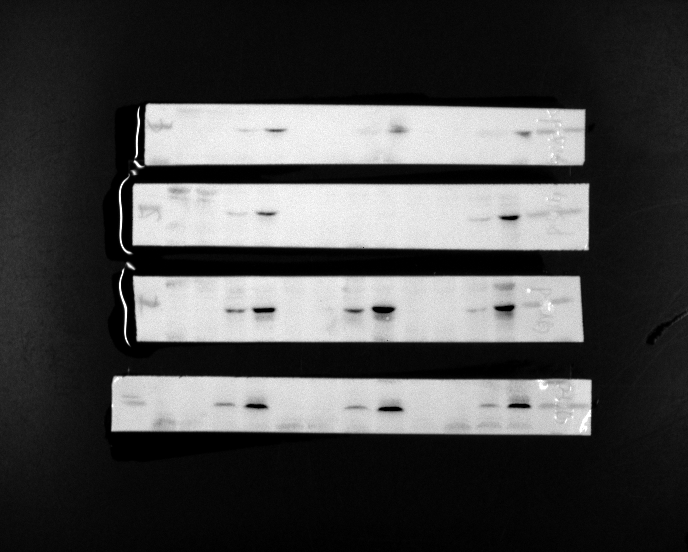

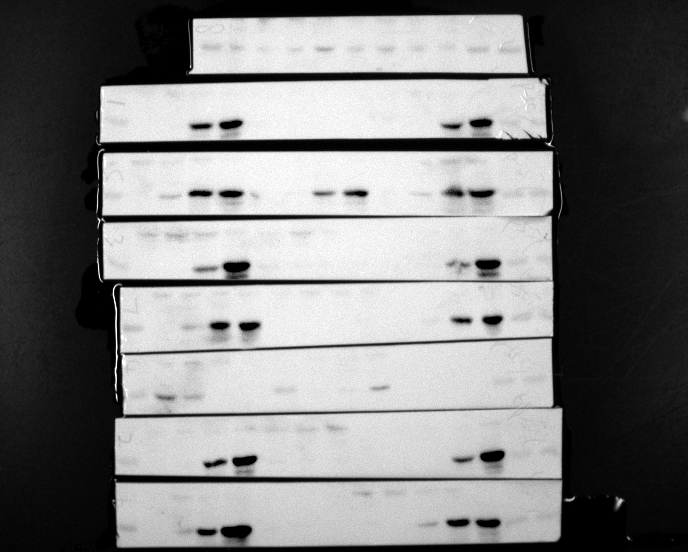

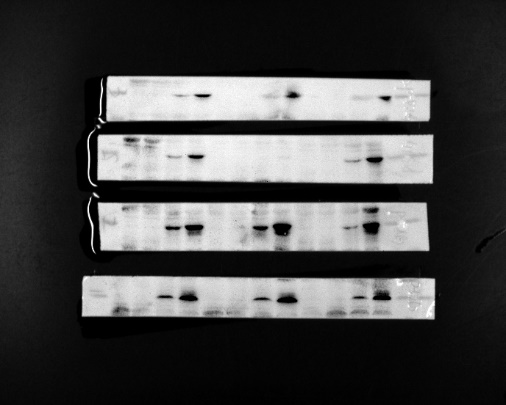

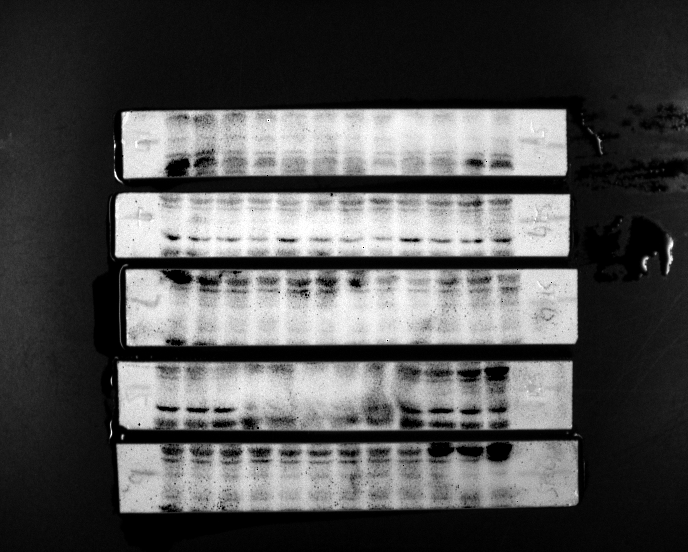

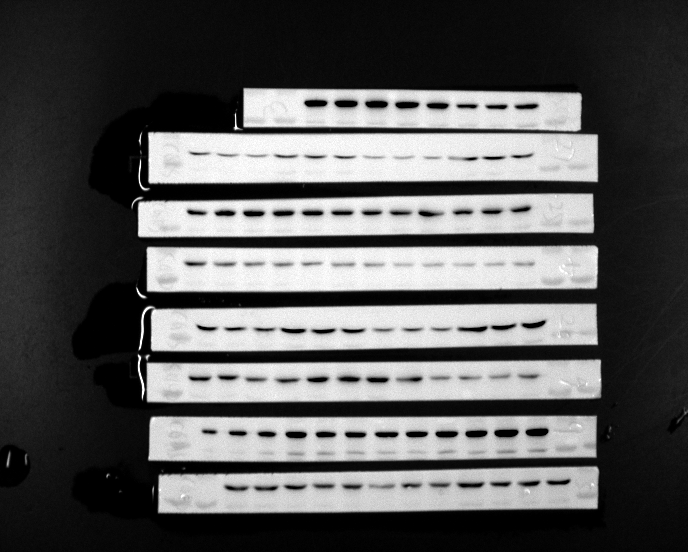


STING

pTBK1

TBK1

cGASme

cGAS

GAPDH

pSTING

Figure 2

A.

pSTING


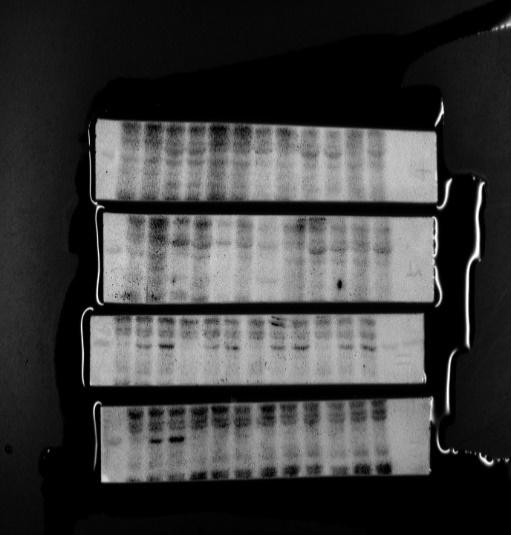

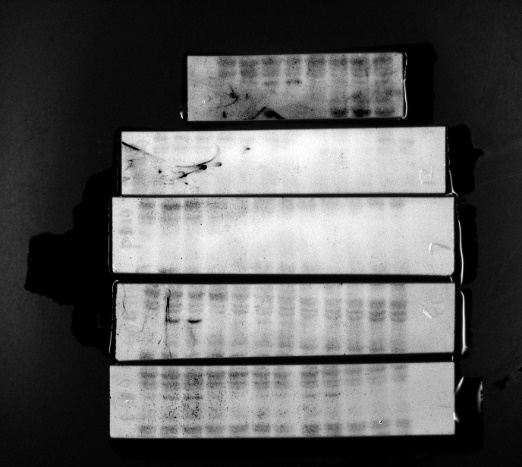

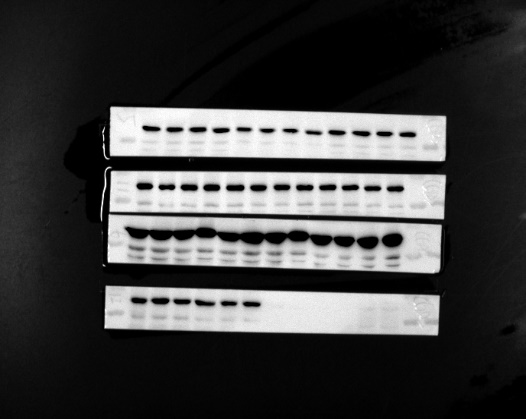

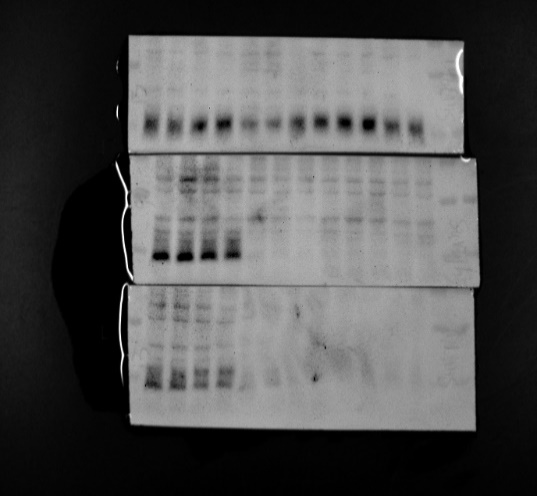

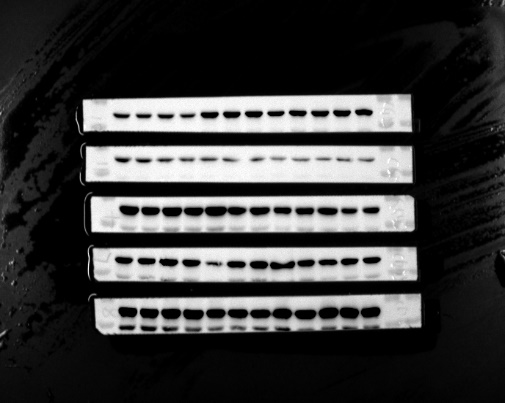

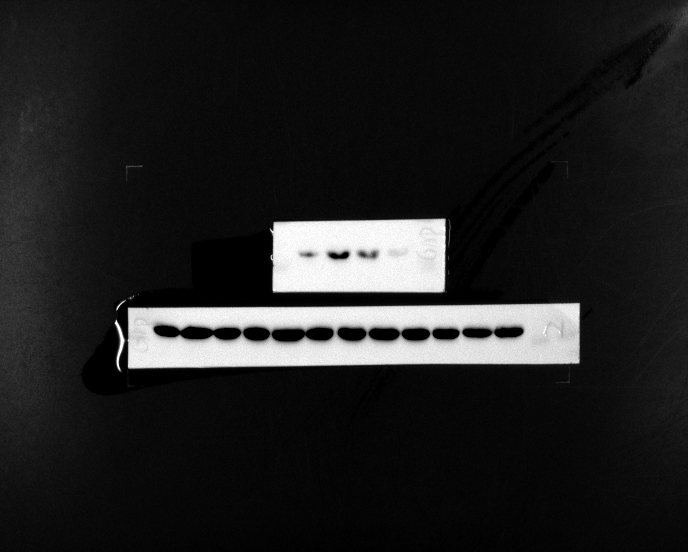

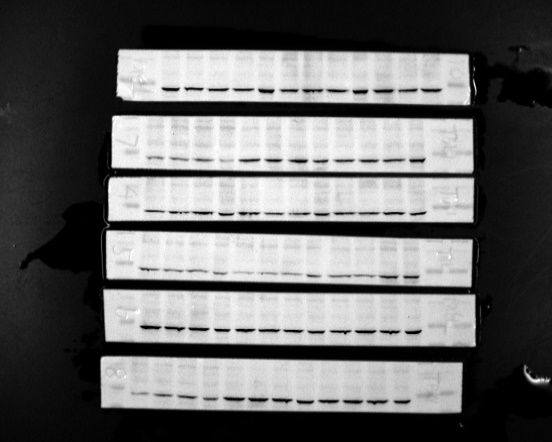

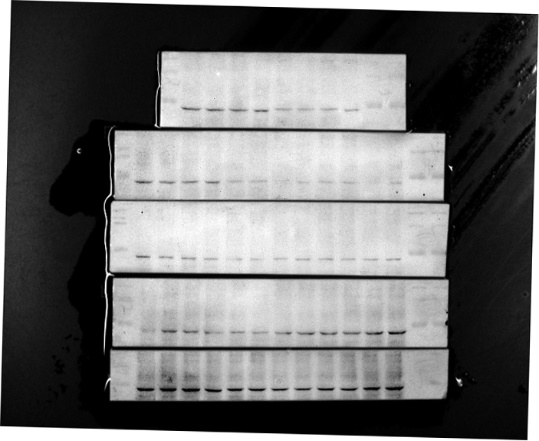


STING

pTBK1

TBK1

cGASme

cGAS

KDM4B

GAPDH

C.


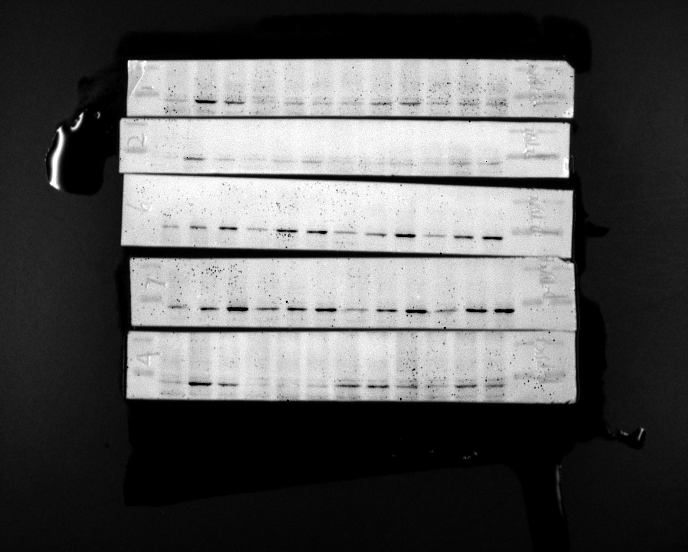

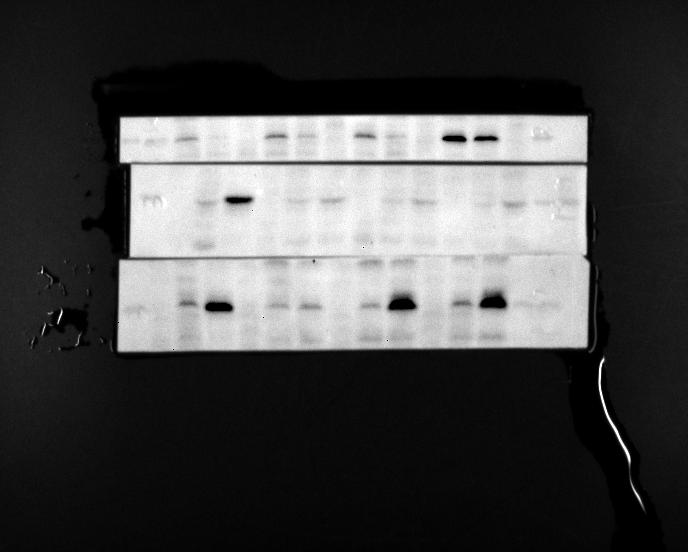

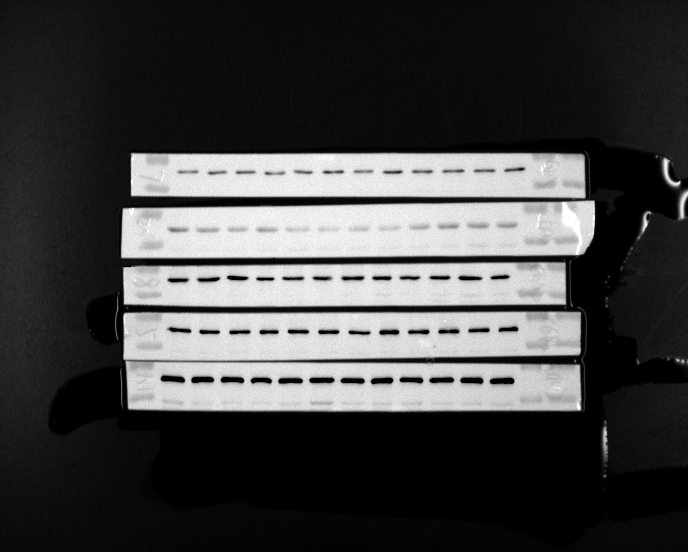

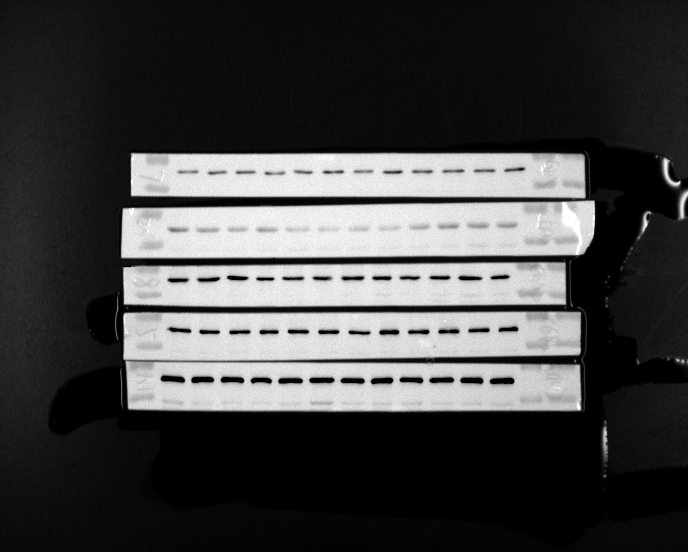

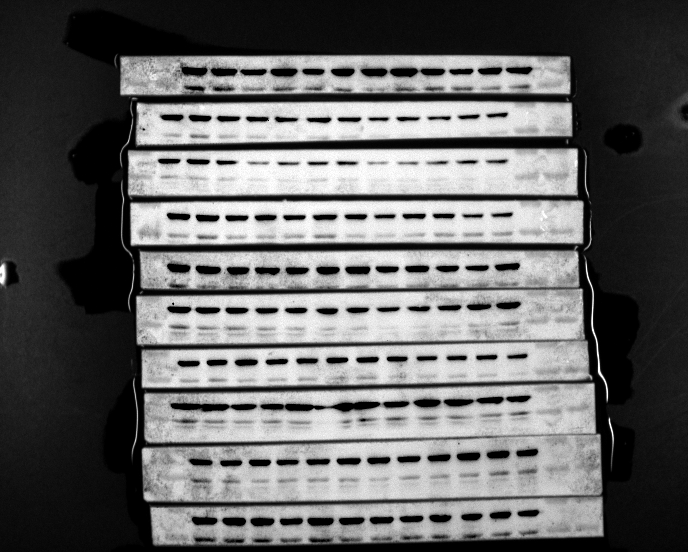

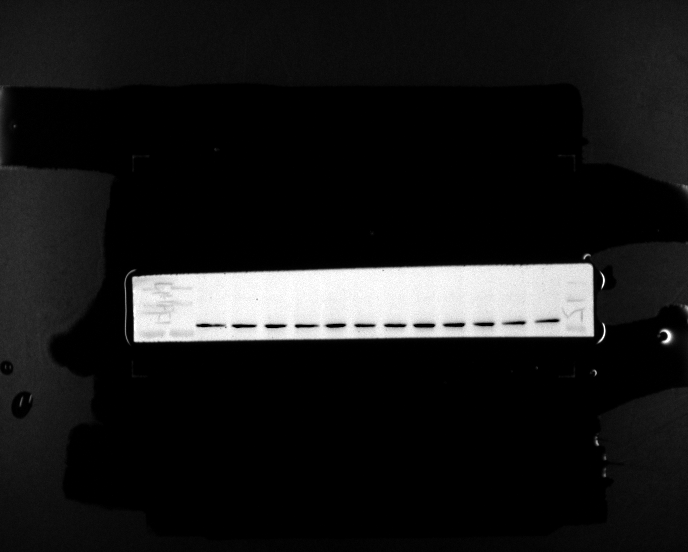

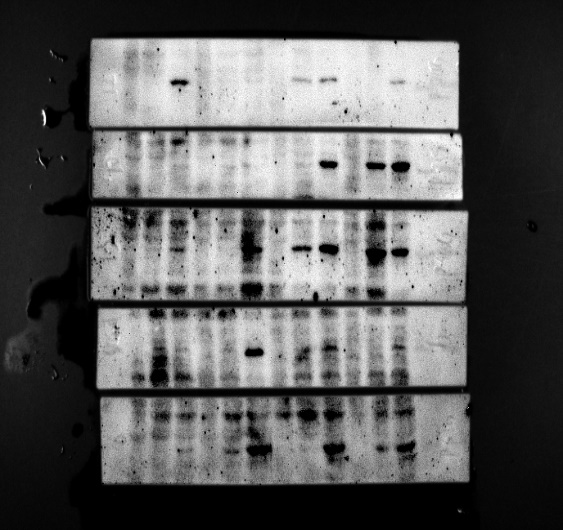

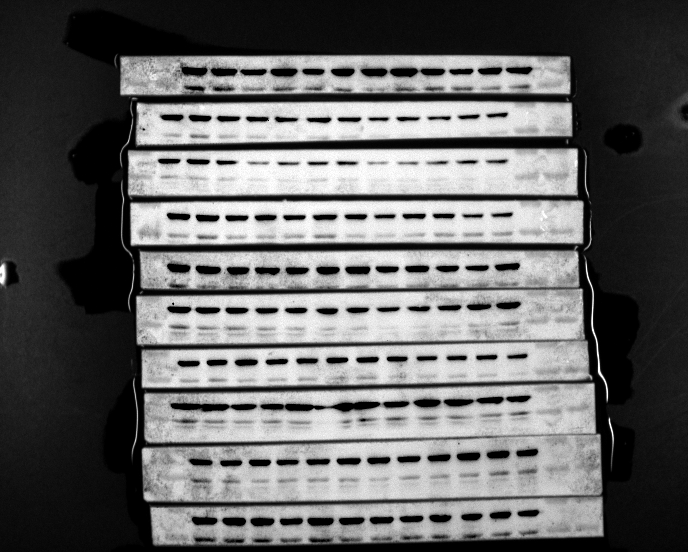

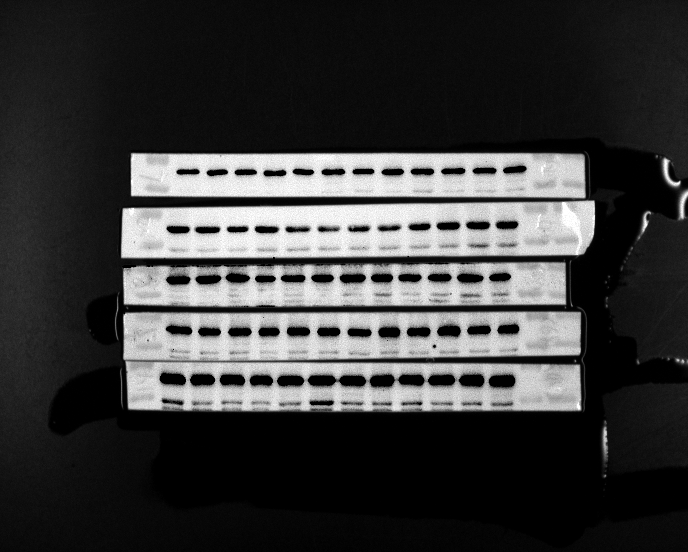

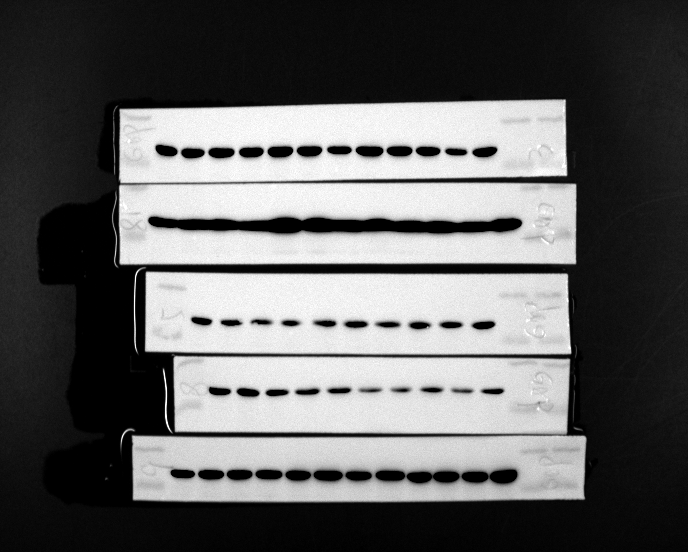

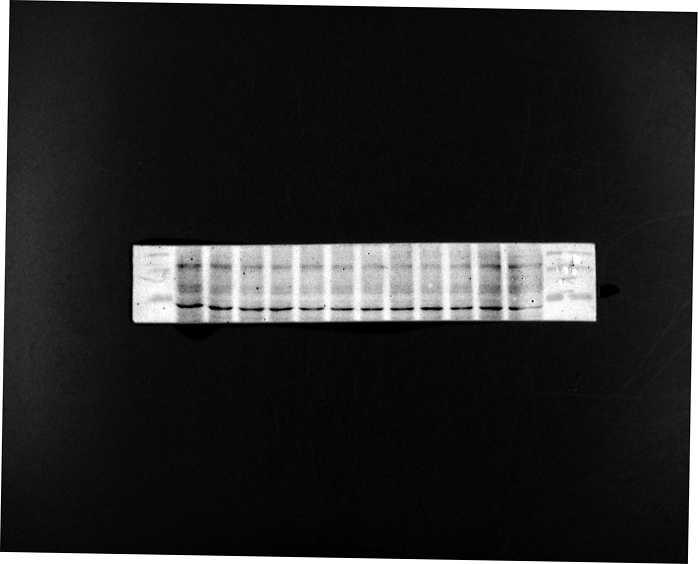

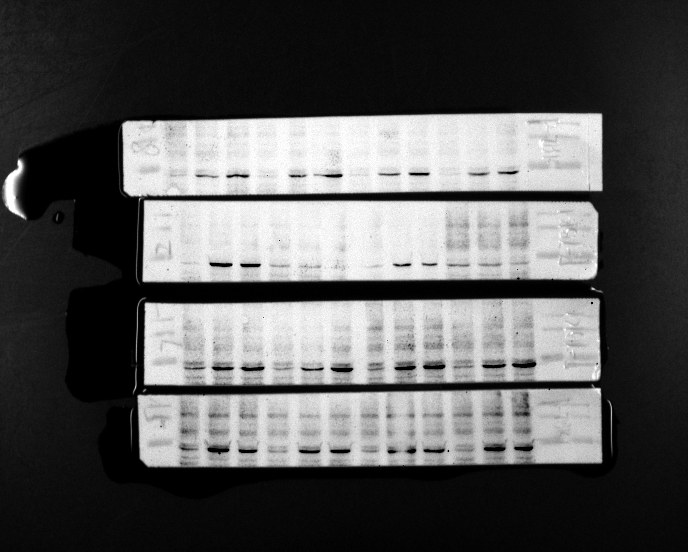

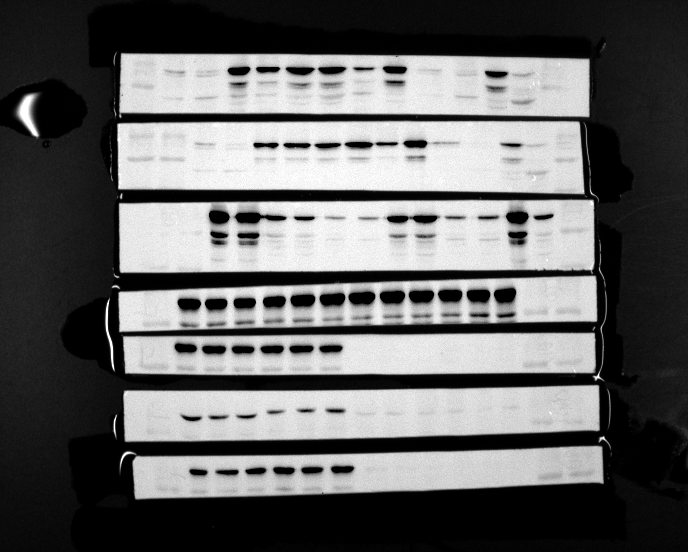

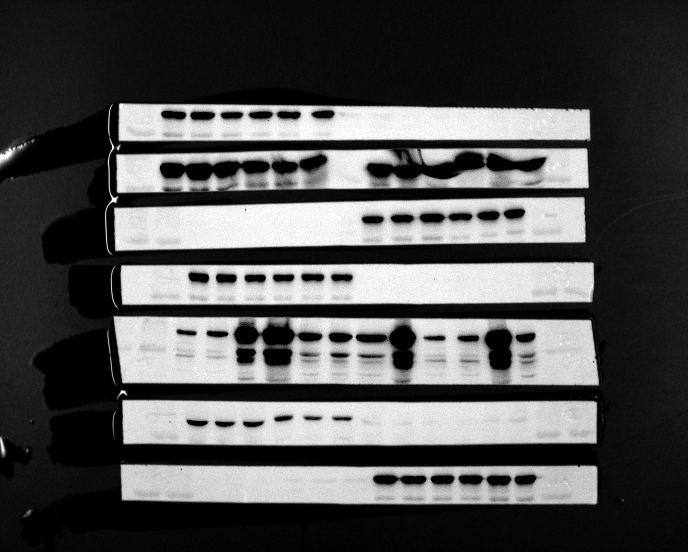


pSTING

STING

pTBK1

TBK1

cGAS

KDM4B

GAPDH

F.


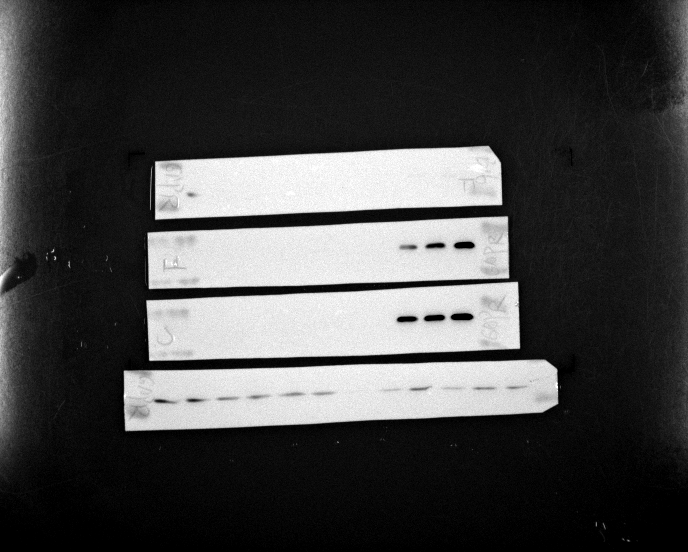

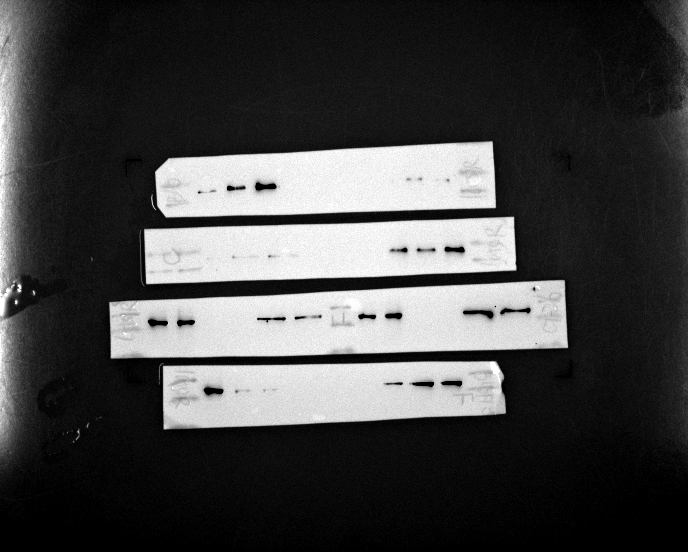

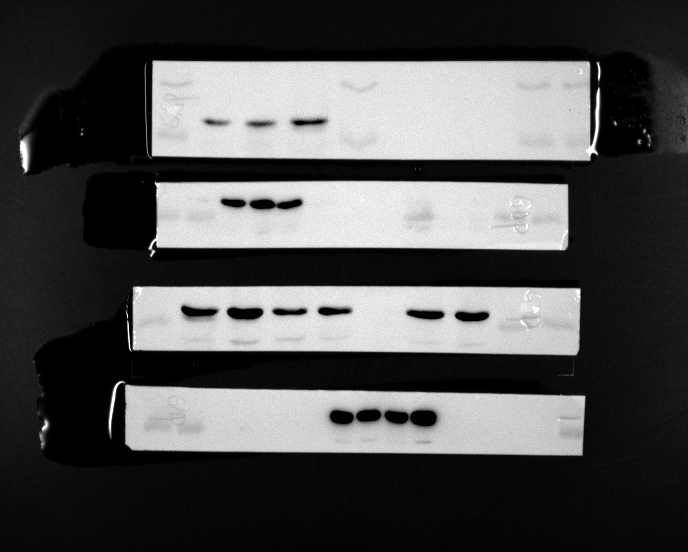

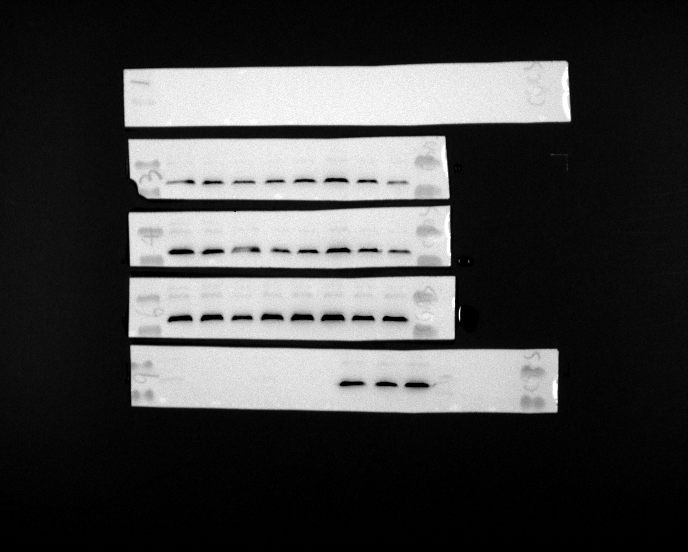

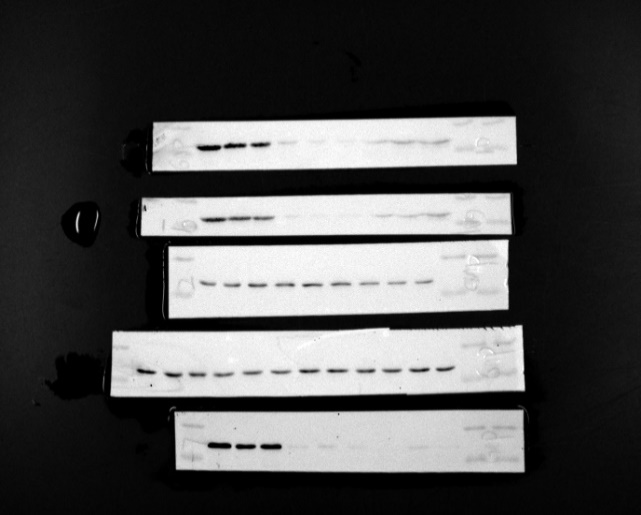


cGASme

KDM4B

GAPDH

CREB

H3

cGAS

Figure 5

A. B. C.

cGASme

Trex1

cGAS

GAPDH

cGAS

GAPDH

cGAS

Trex1

cGAS

cGASme

cGASme

cGAS

Trex1

cGAS

GAPDH

D.

STING

pTBK1

TBK1

cGAS

GAPDH

pSTING

The QPCR original data

Figure 1

C. Three independent experiments

Figure 2

B.

E.

**Figure 4**

C.

D.

E.

Figure 5

E.

F.

**Figure 6**

B

C.

Figure7

C.

F.
